# A New Green Pitviper of the Genus *Trimeresurus* Lacépède, 1804 (Squamata: Viperidae) from Xizang, China †

**DOI:** 10.3390/ani15182675

**Published:** 2025-09-12

**Authors:** Yuhao Xu, Tan Van Nguyen, Zhenqi Wang, Tierui Zhang, Nikolay A. Poyarkov, Cong Wei, Gernot Vogel, Jianchuan Li, Jundong Deng, Fanyue Sun, Lifang Peng, Shiyang Weng

**Affiliations:** 1 State Key Laboratory of Plateau Ecology and Agriculture, Qinghai University, Xining 810016, China; yuhao_xu@sinoophis.com (Y.X.);; 2 Institute of Plateau Biology of Xizang Autonomous Region, Lhasa 850008, China; 3 The School of Medicine & Pharmacy, Duy Tan University, Da Nang 550000, Vietnam; tan.sifasv@gmail.com; 4 Center for Entomology & Parasitology Research, Duy Tan University, Da Nang 550000, Vietnam; 5 The Co-Innovation Center for Sustainable Forestry in Southern China, College of Biology and the Environment, Nanjing Forestry University, Nanjing 210037, China; 6 The Anhui Provincial Key Laboratory of Biodiversity Conservation and Ecological Security in the Yangtze River Basin, College of Life Sciences, Anhui Normal University, Wuhu 241000, China; 7 Department of Vertebrate Zoology, Lomonosov Moscow State University, Leninskiye Gory, GSP–1, Moscow 119991, Russia; 8 Society for South East Asian Herpetology, Im Sand-3, D-69115 Heidelberg, Germany; 9 Xizang Museum of Natural Science, Lhasa 850011, China

**Keywords:** *Viridovipera*, morphology, taxonomy, molecular phylogeny, systematics, Himalaya

## Abstract

Species diversity within the green pit vipers of the genus *Trimeresurus* is likely underestimated. In this study, we describe a new species of *Trimeresurus* from the Xizang Autonomous Region, China, based on both morphological and molecular evidence. The new species, *Trimeresurus pretiosus* sp. nov., is assigned to the subgenus *Viridovipera* Malhotra Thorpe. It is distinguished from its congeners by the following combination of morphological characters: (1) first supralabial completely separated from nasal scale; (2) hemipenis short and spinose, reaching the 12th subcaudal when fully everted; (3) small adult size, with a maximum known snout-vent length of 516 mm in males and 512 mm in females; (4) dorsal scales in 19–19–15 rows, weakly keeled except for the outermost rows; (5) 140–143 ventral scales; (6) 56–58 subcaudal scales in males and 54 in females, partially arranged in a single row; (7) iris reddish-brown in males, orange-yellow in females; (8) body uniformly bright grass-green; postocular streak absent or faint white in males, absent in females; (9) ventrolateral stripe consisting of red above and white below and wide in males, only white and narrow in females; (10) ventral surfaces greenish-yellow; (11) tail distinctly reddish-brown dorsally, with the colouration extending from tail base to tip. Molecular analyses based on the mitochondrial *16S*, cyt *b*, and *ND4* gene fragments indicate that the new species is genetically divergent from all congeners, with uncorrected p-distances ranging from 5.8% to 12.5% in the cyt *b* gene and from 6.7% to 11.0% in the *ND4* gene. The new species is currently known only from the southern slopes of the central Himalayas in Yadong County, and it represents the second known *Viridovipera* species from the southern Himalayas. With this discovery, the total number of snake species known from the Xizang Autonomous Region increases to 67, underscoring the importance of continued herpetofaunal surveys in this biogeographically complex region.

## Simple Summary

We report the discovery of a western record of green pit viper (*Trimeresurus*, subgenus *Viridovipera*) from Yadong County, Xigaze City, Xizang Autonomous Region, China. This population shows notable genetic divergence in the mitochondrial 16S ribosomal RNA (*16S*), cytochrome *b* (cyt *b*), and NADH dehydrogenase subunit 4 (*ND4*) gene fragments, with uncorrected cyt *b* distances of ≥5.8% from its closest relative, *T.* cf. *medoensis*, and ≥6.2% from other congeners in the subgenus *Viridovipera*, as well as *ND4* distances of 7.8% from *T.* cf. *medoensis* and ≥6.7% from other congeners. In addition to genetic distinctiveness, it can be reliably distinguished from other congeners by several key morphological characteristics. Based on the combined molecular and morphological evidence, we describe this population as a new species, *Trimeresurus pretiosus* sp. nov. This finding raises the total number of known *Trimeresurus* species to 56, including 15 species reported from China. In addition, our discovery highlights the Himalayan region as a global hotspot for pitviper diversity and endemism, emphasising its importance for future taxonomic and conservation studies.

## 1. Introduction

The genus *Trimeresurus* Lacépède, 1804, represents a prominent clade of Asian pit vipers that are both taxonomically rich and of considerable medical significance. Currently, 55 valid species are recognised within the genus [1,2,3,4,5,6,7], and its members are widely distributed across South, Southeast, and East Asia from northwestern India and Nepal to southern China, through mainland and insular Southeast Asia as far as Timor and the Lesser Sunda Islands [8,9,10]. Despite numerous taxonomic revisions in recent years, the diversity within *Trimeresurus* is still not fully resolved [3,4,5,6,7]. This is primarily due to high morphological similarity among species, which complicates diagnosis and hinders the discovery of cryptic lineages [3,4,5,6,11,12].

According to the classification proposed by Mirza et al. [1], the genus is currently divided into six subgenera: *Trimeresurus* s. str., *Parias* Gray, 1849, *Popeia* Malhotra and Thorpe, 2004, *Himalayophis* Malhotra and Thorpe, 2004, *Sinovipera* Guo and Wang, 2011, and *Viridovipera* Malhotra and Thorpe, 2002 (see also Idiiatullina et al. [5]; Pawangkhanant et al. [7]). Species placed within *Viridovipera* are defined by two key morphological features: the first supralabial scale is completely separated from the nasal scale, and the hemipenis is short and spinose [1,13]. Currently, seven nominal species are assigned to this subgenus, including *Trimeresurus gumprechti* David, Vogel, Pauwels and Vidal, 2002 [type locality: Phu Luang Wildlife Research Station, Loei Province, Thailand], *T*. *mayaae* Rathee, Purkayastha, Lalremsanga, Dalal, Biakzuala, Muansanga and Mirza, 2022 [type locality: Champhai District, Mizoram State, India], *T*. *medoensis* Zhao, 1977 [type locality: Motuo (Medog) County, Xizang Autonomous Region, China]; *T*. *stejnegeri* Schmidt, 1925 [type locality: Shaowu, Fujian Province, China]; *T*. *truongsonensis* Orlov, Ryabov, Bui and Ho, 2004 [type locality: Phong Nha-Ke Bang National Park, Quang Binh Province, Vietnam], *T*. *vogeli* David, Vidal and Pauwels, 2001 [type locality: Khao Yai National Park, Nakhon Ratchasima Province, Thailand]; and *T*. *yunnanensis* Schmidt, 1825 [type locality: Tengchong, Yunnan Province, China] [11,14].

Characterised by extreme elevation gradients, complex topography, and a wide range of microclimates, the Himalayan region harbours exceptional biodiversity and a high degree of endemism. These features, combined with its role as both a biogeographic barrier and corridor, have led to its recognition as a global biodiversity hotspot [15,16]. However, due to its remoteness, rugged terrain, and frequent geological instability, many parts of the Himalayas remain poorly surveyed, and the species diversity is still not well understood. To help address this knowledge gap, as part of ongoing herpetofaunal surveys in the central Himalayas, three specimens of green pitvipers assignable to the subgenus *Viridovipera* were collected in June 2025 from Yadong County, Xigaze City, Xizang Autonomous Region, China. A detailed morphological assessment and mitochondrial DNA analysis indicate that these individuals represent an undescribed species. In this paper, we formally describe this population as a new species of *Trimeresurus*.

## 2. Materials and Methods

### 2.1. Sample Collection

**Figure 1:**
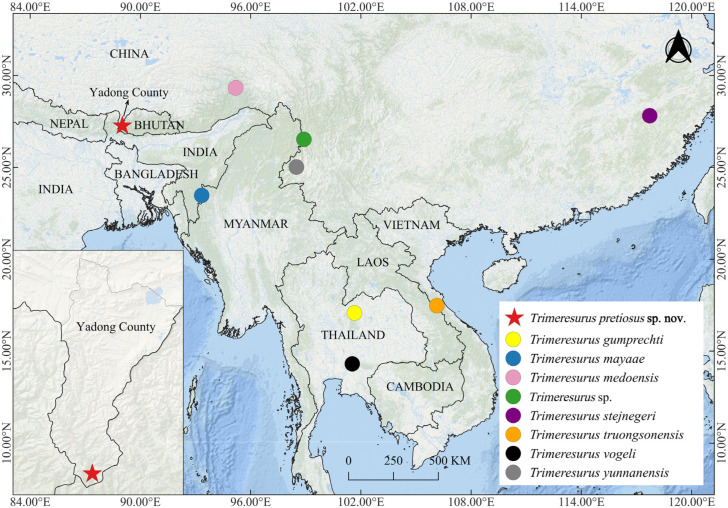
Map showing the type localities of the new species and other *Trimeresurus* species of the subgenus *Viridovipera*. Red star: *Trimeresurus pretiosus* sp. nov. from Xiayadong, Yadong, Xizang, China; yellow circle: *T. gumprechti* from Phu Luang Wildlife Research Station, Loei, Thailand; blue circle: *T*. *mayaae* from Champhai, Mizoram, India; pink circle: *T*. *medoensis* from Motuo (Medog), Xizang, China; green circle: *Trimeresurus* sp. from Fugong, Yunnan, China; purple circle: *T*. *stejnegeri* from Shaowu, Fujian, China; orange circle: *T*. *truongsonensis* from Phong Nha-Ke Bang NP, Quang Binh, Vietnam; black circle: *T*. *vogeli* from Khao Yai NP, Nakhon Ratchasima, Thailand; and grey circle: *T*. *yunnanensis* from Tengchong, Yunnan, China.

Fieldwork was conducted in forested areas of Yadong County, Xigaze City, Xizang Autonomous Region, China, in June 2025 (Figure 1). Geographic coordinates and elevation were recorded using the TwoStep Outdoor Assistant v7.9.13 (Shenzhen 2bulu Information Technology Co., Ltd., Shenzhen, China). A total of five individuals were encountered during the field survey, of which two adult males and one adult female were collected. These specimens were located during afternoon excursions, collected using snake hooks, photographed in life, and humanely euthanised with a buffered MS-222 (tricaine methanesulfonate) solution. Subsequently, the specimens were fixed in 10% formalin and transferred to 75% ethanol for long-term preservation. Liver tissue samples were collected fresh, preserved in 95% ethanol, and stored at −20 °C for subsequent molecular analyses. All voucher specimens were deposited in the herpetological collection of Qinghai University, Qinghai Province, China (QHU). All procedures followed the regulations of the Wildlife Protection Law of China and were approved by the Institutional Ethics Committee of Qinghai University (Protocol No. PJ202501-89).

### 2.2. Molecular Methods and Phylogenetic Analysis

Total genomic DNA was extracted from the ethanol-preserved liver tissues using the QIAamp DNA Mini Kit (QIAGEN, Changsheng Biotechnology Co., Ltd., Changchun, China). Three mitochondrial DNA (mtDNA) fragments, including 16S ribosomal RNA (*16S*), cytochrome *b* (cyt *b*), and NADH dehydrogenase subunit 4 (*ND4*), were amplified using polymerase chain reaction (PCR). The amplification of 16S was conducted with primers 16S1LM (5′-CCGACTGTTGACCAAAAACAT-3′) and 16SH1 (5′-TCCGGTCTGAACTCAGATCACGTAGG-3′), following the protocol of Nguyen et al. [17]. For cyt *b*, we used primers L14910 (5′-GACCTGTGATMTGAAAACCAYCGTTGT-3′) and H16064 (5′-CTTTGGTTTACAAGAACAATGCTTTA-3′), as described by Burbrink et al. [18]. The ND4 fragment was amplified using Trim-ND4F (5′-CACCTATGACTACCAAAAGCTCATGTAGAGC-3′) and Trim-ND4LEUR (5′-CATTACTTTTACTTGGATTTGCACCA-3′), following Salvi et al. [19]. The PCR products were sequenced by Shanghai Map Biotech Co., Ltd. (Shanghai, China). Raw sequences were assembled using SeqMan (DNASTAR) [20] and newly generated sequences were submitted to GenBank (Table 1).

For phylogenetic analysis, a total of 193 sequences were included (Table 1), of which 23 were newly generated in this study and 170 were retrieved from GenBank. These included representatives from 32 species of *Trimeresurus* and two outgroup taxa: *Craspedocephalus puniceus* (Boie, 1827) and *Peltopelor malabaricus* (Jerdon, 1854). Sequences were aligned in MEGA X [34]. The Maximum Likelihood (ML) analysis was conducted in IQ-TREE v1.6.12 [35] using the best-fit model GTR + F + I + G4 for all three fragments (*16S*, cyt *b*, and *ND4*), as determined by ModelFinder for IQ-Tree in PhyloSuite 1.2.3 according to Bayesian Information Criterion (BIC) [36,37]. Node support was assessed using both the Ultrafast Bootstrap Approximation (UFB) and the SH-like approximate likelihood ratio test (SH). UFB values were calculated with 5000 bootstrap replicates, with values ≥ 95% considered strong support [38]. SH was conducted with 1000 replicates, and values ≥ 80% were regarded as well supported [39]. The Bayesian Inference (BI) analysis was conducted via MrBayes v3.2.7a [40] under the best-fit model GTR + F + I + G4 for all three fragments (*16S*, cyt *b*, and *ND4*), which was calculated according to BIC as well by ModelFinder for MrBayes in PhyloSuite 1.2.3 [37]. In the BI analysis, three independent runs were conducted with 1 × 10^7^ generations and sampled every 1000 generations, with the first 25% of samples discarded as burn-in. Nodes with Bayesian posterior probabilities (PP) ≥ 0.95 were considered strongly supported [41]. Uncorrected pairwise genetic distances (*p*-distances) among species were also calculated using MEGA X [34].

### 2.3. Morphological Examination

A total of 45 morphological characteristics were recorded for each specimen (following Vogel et al. [2]; Nguyen et al. [12]). Measurements were taken with a Mitutoyo digital calliper (CD-15AX, Mitutoyo Corporation, Kawasaki, Japan) to the nearest 0.1 mm, except for body and tail lengths, which were measured to the nearest millimetre with a measuring tape. The number of ventral scales was counted according to Dowling [42]. Half ventrals were counted as one. The first enlarged shield anterior to the ventrals was regarded as a preventral and was present in all examined specimens. The first scale under the tail meeting its opposite was regarded as the first subcaudal, and the terminal scute was not included in the number of subcaudals. The dorsal scale rows were counted at one head length behind the head, at midbody, and at one head length before the vent. In the number of supralabials touching the subocular, those only touching the presubocular were not included. Infralabials were considered to be those shields that were completely below a supralabial and bordering the mouth gap. The first sublabial was defined as the scale that starts between the posterior chin shield and the infralabials and that borders the infralabials. Values for paired head characters were recorded on both sides of the head and were reported in a left-right order. In addition, we paid special attention to diagnostic colour pattern characters, such as eye colour, postocular streak, ventrolateral stripe, tail colouration, and overall body colouration. The sex was determined by dissecting the ventral base of the tail and examining for the presence of hemipenes.

Abbreviations. Morphological descriptions and morphometry follow standardised abbreviations revised according to Darko et al. [43]. The morphometric characters are as follows: **SVL** = snout-vent length; **TAL** = tail length; **TL** = total length; **TAL/TL** = ratio of tail length to total length; **HL** = head length; **HW** = head width; **ESD** = eye-snout distance, measured from the tip of the snout to the anterior edge of the eye; **ED** = eye diameter; **EN** = eye-to-nostril distance, measured from the anterior margin of the eye to the posterior margin of the nostril; **SOL** = subocular length; and **SOW** = subocular width.

The scalation characters are as follows: **VS** = number of ventral scales; **SC** = number of subcaudal scales; **CP** = cloacal plate; **SL** = supralabials; **IL** = infralabials; **DSR** = dorsal scale rows; **ASR** = anterior dorsal scale rows; **MSR** = dorsal scale rows at midbody; **PSR** = posterior dorsal scale rows; **PRO** = preoculars; **PO** = postoculars; **SO** = supraoculars; **SBO** = suboculars; **IOS** = interorbital scales, number of scales at the narrowest point between the orbits; **SpOC** = number of dorsal head scales surrounding the supraocular; **Nasal**; **INS** = number of scales separating the internasals; and **TEMP-keeled** = temporal region keeled or not.

Other abbreviations: **AR** = Autonomous Region; **NP** = National Park; **NR** = Nature Reserve; **Mt** = Mountains; **a.s.l.** = above sea level.

## 3. Results

### 3.1. Phylogenetic Relationships

**Figure 2:**
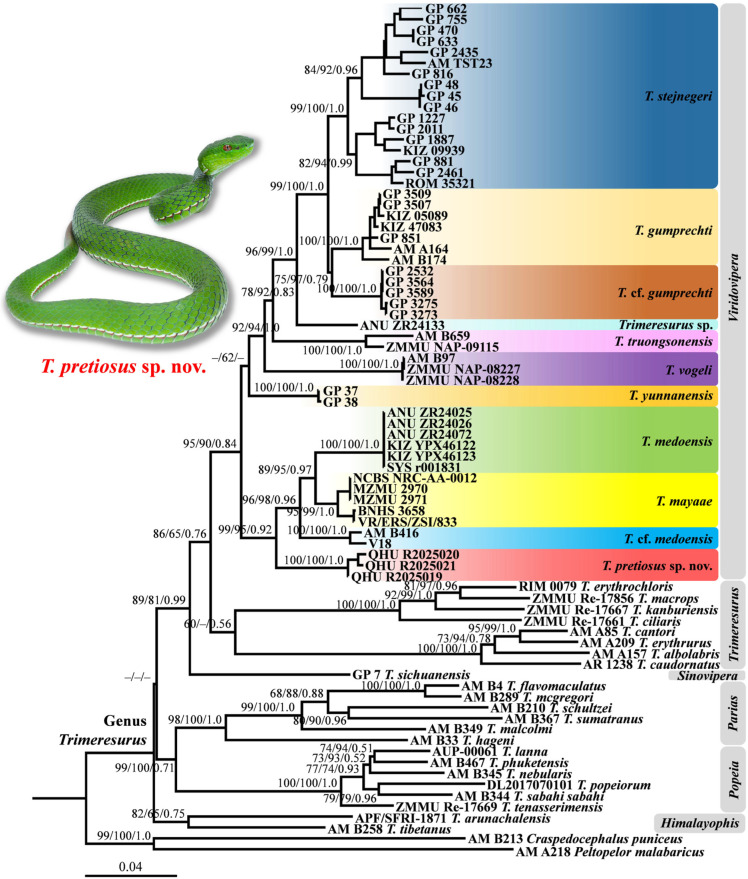
Phylogram of the genus *Trimeresurus* inferred from three mitochondrial (*16S*/cyt *b*/*ND4*) fragments. The branch support values are presented with the SH-like approximate likelihood ratio test (SH)/Ultrafast Bootstrap Approximation (UFB)/Bayesian posterior probabilities (PP); the ones lower than 50 or 0.5 are displayed as “–”. Photograph on thumbnail by Y.H. Xu.

The concatenated sequence alignment was 2314 bp in length (*16S* = 508 bp; cyt *b* = 1034 bp; and *ND4* = 772 bp). Both ML and BI analyses yielded congruent topologies (Figure 2). Our mtDNA-based phylogeny recovered all sampled *Trimeresurus* species within a single clade. However, the relationships among species within the genus remained incompletely resolved. The monophyly of the subgenera *Parias* and *Popeia* was strongly supported (SH 98/UFB 100/PP 1.0; SH 100/UFB 100/PP 1.0 respectively), while subgenus *Himalayophis* and *Viridovipera* received moderate support (SH 82/UFB 65/PP 0.75; SH 95/UFB 90/PP 0.84, respectively). The subgenus *Trimeresurus* was recovered as monophyletic, but with very low support (SH 60; UFB –; PP 0.56), leaving the true relationships among these clades unresolved.

Within *Viridovipera*, interspecific relationships were generally well resolved (Figure 2). Two specimens (AM B416 from Northern Myanmar and V18 from Northeast India) previously identified as *T. medoensis* formed a distinct lineage sister to the *T. medoensis*–*T. mayaae* clade and were provisionally referred to as *T.* cf. *medoensis*. Three specimens from Yadong County, Xizang AR, China, constituted a separate, well-supported lineage (SH 99/UFB 95/PP 0.92) sister to the clade comprising *T. mayaae*, *T. medoensis*, and *T.* cf. *medoensis*. The remaining species (*T. gumprechti*, *T. truongsonensis*, *T. vogeli*, *T. stejnegeri*, and *T. yunnanensis*), clustered into another lineage with low branch support values (SH –/UFB 62/PP –). Additionally, a newly included specimen (ANU ZR24133) from Fugong County, Yunnan Province, China, formed an independent lineage near the clade of *T. gumprechti* and *T. stejnegeri*, showing uncorrected *p*-distances of 5.6–9.6% in the cyt *b* gene and 4.9–9.4% in the *ND4* gene from other congeners, and was provisionally referred herein to as *Trimeresurus* sp.

Uncorrected *p*-distances for the cyt *b* and *ND4* gene fragments are summarised in Table 2 and Table 3, respectively. Interspecific genetic distances within *Viridovipera* ranged from 4.4% (*T. mayaae* and *T. medoensis*) to 12.8% (*T. stejnegeri* and *T. truongsonensis*) for the cyt *b* gene (Table 2), and from 2.9% (*T. gumprechti* and *T.* cf. *gumprechti*) to 12.1% (*T.* cf. *medoensis* and *T. vogeli*) for the *ND4* gene (Table 3). The Yadong population displayed substantial genetic divergence from all congeners, ranging from 5.8% (vs. *T.* cf. *medoensis*) to 12.5% (vs. *T. stejnegeri*) for the cyt *b* gene, and from 6.7% (vs. *T. yunnanensis*) to 11.0% (vs. *T. vogeli*) for the *ND4* gene. Given its distinct phylogenetic placement, marked genetic differentiation, and consistent morphological distinctiveness, we recognise this population as a new species, and formally describe it below.

### 3.2. Taxonomy

*Trimeresurus pretiosus* Xu, Nguyen, Wang, Zhang, Poyarkov, Wei, Vogel, Peng, and Weng sp. nov.

Holotype. QHU R2025019, adult male, collected from Xiayadong Township, Yadong County, Xigaze City, Xizang Autonomous Region, China (27.262° N, 89.016° E; elevation 1824 m a.s.l.), collected by Zhenqi Wang, Yuhao Xu, Fanyue Sun, and Lifang Peng on 22 June 2025.

Paratypes (n = 2). QHU R2025020 (adult male) and QHU R2025021 (adult female), collected by Zhenqi Wang, Yuhao Xu, Fanyue Sun, and Lifang Peng on 23 June, with the same collection information as the holotype.

Diagnosis. *Trimeresurus pretiosus* sp. nov. is distinguished from all of its congeners by a combination of the following morphological characters: (1) first supralabial completely separated from nasal scale; (2) hemipenis short and spinose, reaching 12th subcaudal when fully everted; (3) adult body size relatively small, with a maximum known snout-vent length of 516 mm in males and 512 mm in females; (4) dorsal scales in 19–19–15 rows, weakly keeled except for the outermost rows; (5) 140–143 ventral scales; (6) 56–58 subcaudal scales in males and 54 in females, partially arranged in a single row; (7) iris reddish-brown in males, orange-yellow in females; (8) body uniformly bright grass-green; postocular streak absent or faint white in males, absent in females; (9) ventrolateral stripe consisting of red above and white below and wide in males; white only and narrow in females; (10) ventral surfaces greenish-yellow; (11) tail distinctly reddish-brown dorsally, with the colouration extending from tail base to tail tip.

Etymology. The specific epithet “*pretiosus*” is a Latin adjective in the nominative case (masculine gender, singular), meaning “precious” or “valuable”. The name refers to the species’ striking and vivid colouration, which makes it resemble a hidden green gem within the pristine forest. Furthermore, although herpetologists have conducted numerous field surveys in Yadong County, Xizang AR, China, for several decades, no green pit viper has ever been recorded from the area until now. The new species represents the currently known westernmost distribution of the subgenus *Viridovipera* and is extremely rare in the wild. Therefore, through the specific epithet, we hope to raise awareness not only of the new species but also of the rich biodiversity of Yadong County, thereby promoting greater attention to and protection of its unique ecological environment. Based on its type locality, Yadong County, we recommend “Yadong Green Pit Viper” as the common English name, “亚东竹叶青蛇” (Yă Dōng Zhú Yè Qīng Shé) as the Chinese name, “Rắn lục xanh báu vật” as the Vietnamese name, “Драгoценная бамбукoвая куфия” (Dragotsennaya bambukovaya kufiya) as the common name in Russian, and “Yadong-Grubenotter” as the common name in German.

**Figure 3:**
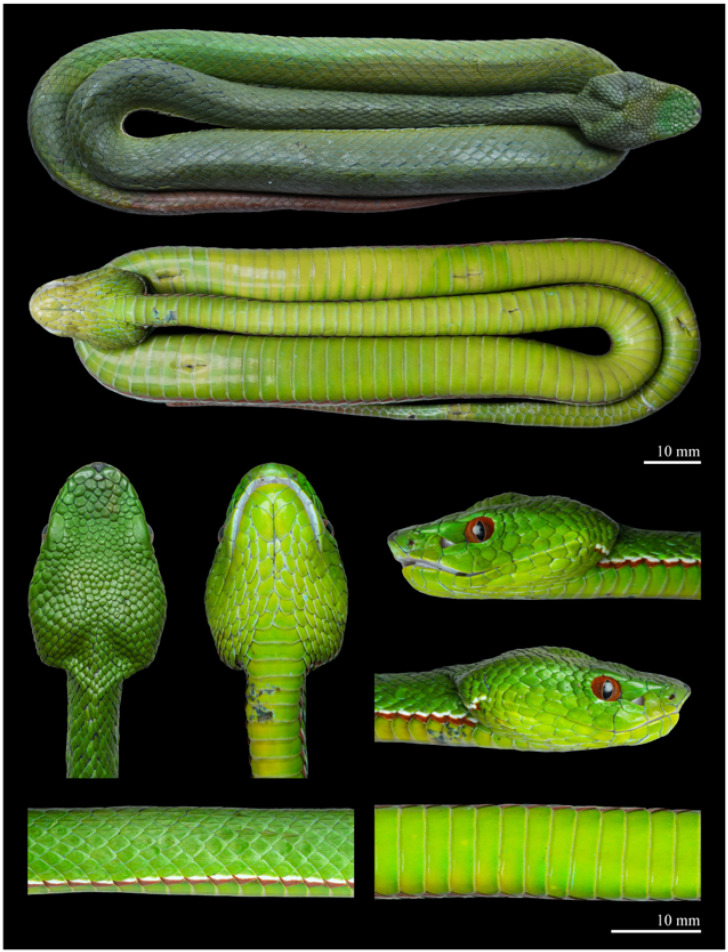
Fresh specimen of the holotype of *Trimeresurus pretiosus* sp. nov. (QHU 2025019, adult male). Photographs by Y.H. Xu. Scale bars = 10 mm.

Description of holotype (Figure 3). *Morphology*. Adult male, in a good state of preservation. Body cylindrical and elongated (SVL 451 mm, TAL 108 mm, and TL 559 mm); tail relatively long (TAL/TL ratio 0.19); head triangular, wide at base, clearly distinct from the neck (HL 24.2 mm, HW 16.1 mm, HW/HL ratio 0.67). Snout elongate, round anteriorly (ESD 6.7 mm, EN 5.8 mm, ESD/HL ratio 0.28). Eyes of moderate size (ED 3.8 mm); the pupil vertically elliptical.

Head scalation. Rostral triangular (width 3.5 mm, height 3.1 mm), curved onto the dorsal surface of the snout, clearly visible from above; nasal single, undivided, completely separated from the first supralabial; nostril positioned centrally within the nasal scale; a pair of distinctly enlarged internasals, separated from each other by the rostral and small scale positioned directly behind it; 3/4 canthal scales bordering the canthus rostralis between the internasal and corresponding supraocular. Loreal pit present, triangular in shape, located closer to eye than to nostril. PRO 3/3, elongate, two upper preoculars positioned above the loreal pit, both in contact with the single loreal, the lower preocular forms the lower margin of the loreal pit and contacts the 3rd supralabial; PO 2/3; SBO 1/1, long and crescent-like, contacting the 3rd supralabial, separated from the 4th and 5th supralabial by one row of scales and from the 6th supralabial by two scales; SO 1/1, large (SOL 4.7 mm, SOW 2.1 mm, SOW/SOL ratio 0.45); SpOC 8/7. Scales on the dorsal surface of the snout enlarged, flat, smooth, and irregular in shape, gradually decreasing in size posteriorly; cephalic scales small, smooth, and irregularly shaped; IOS 9; temporal and occipital scales very weakly keeled. SL 9/9, the 1st supralabial triangular; the 2nd is the tallest, with a concave upper part forming the anterior border of the loreal pit, contacting the nasal medially but separated in the upper portion by small scale; the 3rd widest, with the remaining supralabials gradually decreasing in size posteriorly; IL 11/10, first pair in contact with each other behind mental; first three pairs in contact with single pair of chin shields.

**Figure 4:**
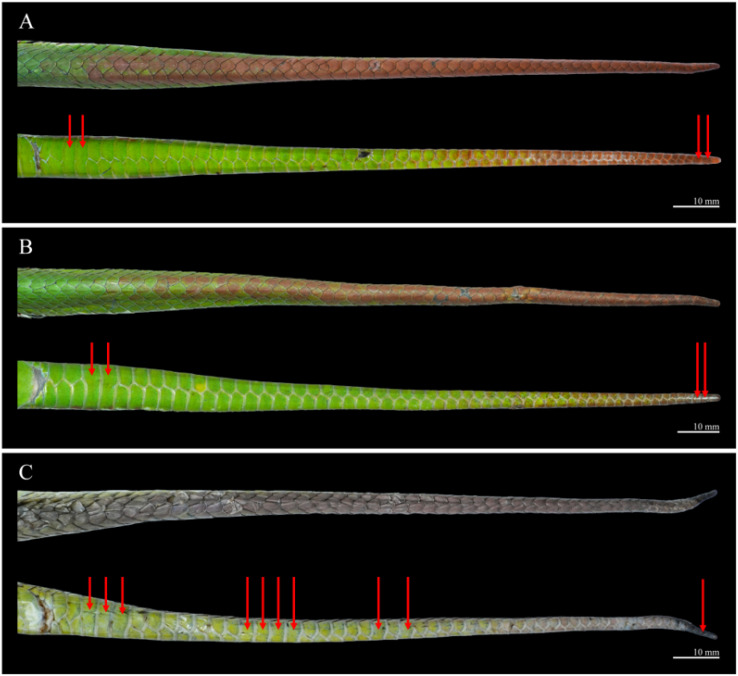
Dorsal and ventral views of the tail of *Trimeresurus pretiosus* sp. nov. Red arrows indicate the subcaudals in a single row. (**A**), QHU R2025019, holotype, adult male; (**B**), QHU R2025020, paratype, adult male; (**C**), QHU R2025021, paratype, adult female. Photographs by Y.H. Xu. Scale bars = 10 mm.

Body scalation. DSR 19–19–15; at midbody, all dorsal scale rows except the outermost weakly keeled, with keels faint laterally and becoming more distinct toward mid-dorsal rows. Outermost row completely smooth. VS 140 (+1 preventrals); SC 58, with the 3rd, 4th, 57th, and 58th single, and all others paired; CP entire (Figure 4).

**Figure 5:**
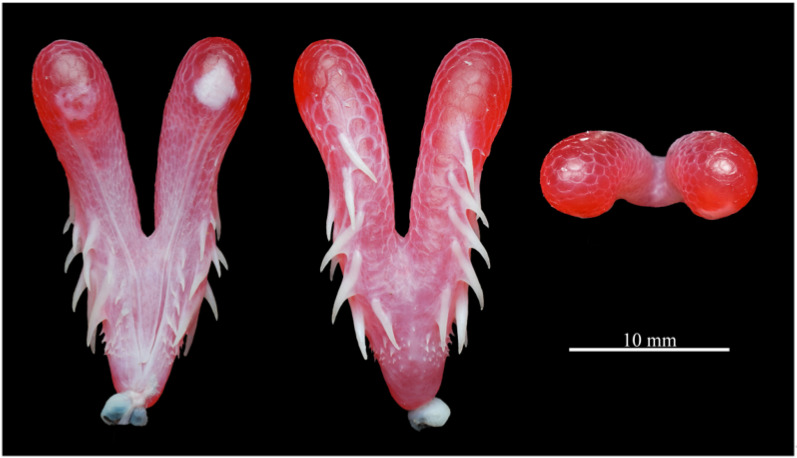
Hemipenis of the holotype of *Trimeresurus pretiosus* sp. nov. (QHU R2025019, adult male). Photographs by Y.H. Xu. Scale bar = 10 mm.

Hemipenis (Figure 5). The description is based on the everted organ of the male holotype (QHU R2025019). Hemipenis relatively short and robust, extending to the level of the 12th subcaudal scale when everted. Total length 23.4 mm; maximum width 13.5 mm. Organ Y-shaped, bilaterally symmetrical, with the bifurcated portion measuring 12.3 mm in length.

Basal one-fifth of truncus smooth. Above this region, sparse, fine spinules appear and gradually increase in size along sulcate side, continuing upward until just below bifurcation point, where they disappear. The lateral surface, at approximately one-fourth of total length from base, armed with a series of long, slender, sharply pointed spines. These spines are arranged in three roughly longitudinal rows: the row nearest to the asulcate surface bearing five spines extending to apical one-fourth; the middle row and the row closer to the sulcate surface each bear three spines extending to about one-half of distal length. A few smaller spines are scattered irregularly distal to these main rows. The entire bifurcated region is covered with weak and smooth calyces. Sulcus spermaticus branches near proximal one-sixth of the organ, running medially along each lobe, and terminating approximately one-fifth from apex. Sulcal lips prominent and well developed.

**Figure 6:**
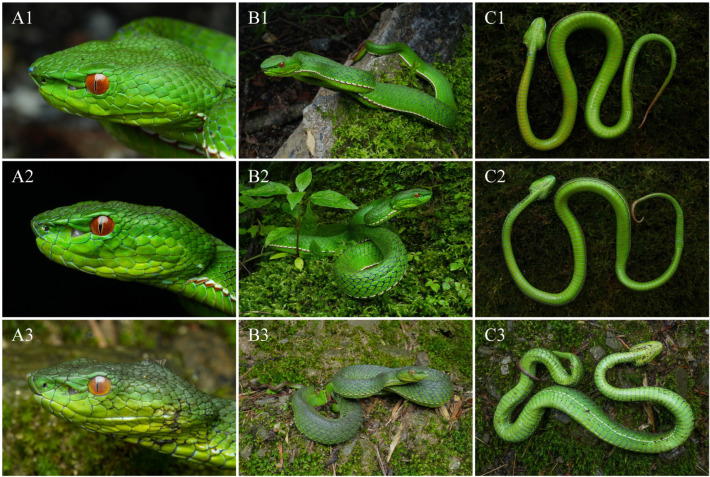
*Trimeresurus pretiosus* sp. nov. in life: lateral views of the head (**A**), dorsal views (**B**), and ventral views (**C**). (**A1**,**B1**,**C1**), QHU R2025019, holotype, adult male; (**A2**,**B2**,**C2**), QHU R2025020, paratype, adult male; (**A3**,**B3**,**C3**), QHU R2025021, paratype, adult female. Photographs by Y.H. Xu.

Colouration of holotype in life (Figure 6(A1,B1,C1)). In life, the dorsal surface of the head and body is uniformly grass-green, lacking postocular streaks or crossbars. The interstitial skin is predominantly black, with irregular grey serrated transverse bands appearing every one to two scales. A distinct bicoloured ventrolateral stripe running along the first dorsal scale row, beginning just behind the corner of the mouth, extending continuously to the to the cloacal region, and then continuing intermittently along the tail to approximately its anterior one-fifth of its length. On each side, the lower third of the first dorsal scale row is light brick red, the middle third white, and the upper portion green. The dorsal surface of the tail is grass-green at the base, overlaid with brick-red blotches mainly distributed across the central region of the anterior half, while the outermost lateral portions remain green. Posteriorly, the blotches expand, rendering the distal half of the tail nearly uniformly brick red. The iris is reddish-brown. The ventral surface of the head and body is uniformly yellowish-green, with the ventral scales faintly edged in brick red. The ventral surface of the tail is also yellowish green, with the margins of the subcaudal scales tinged with brick red in the anterior one-fifth.

Colouration of the holotype in preservative. After one month in preservative, the eyes turned greyish-white, the dorsal colouration darkened to dark green, and the brick-red blotches on the dorsal tail faded to dark reddish-brown or brown. The ventral surface also became noticeably darker.

**Figure 7:**
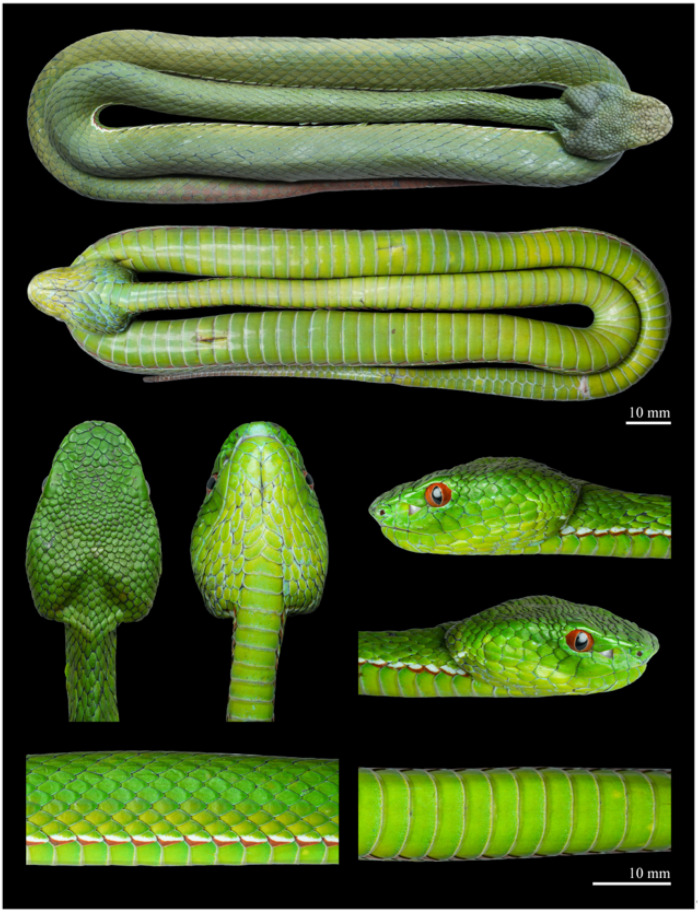
Fresh specimen of the male paratype of *Trimeresurus pretiosus* sp. nov. (QHU R2025020). Photographs by Y.H. Xu. Scale bar = 10 mm.

**Figure 8:**
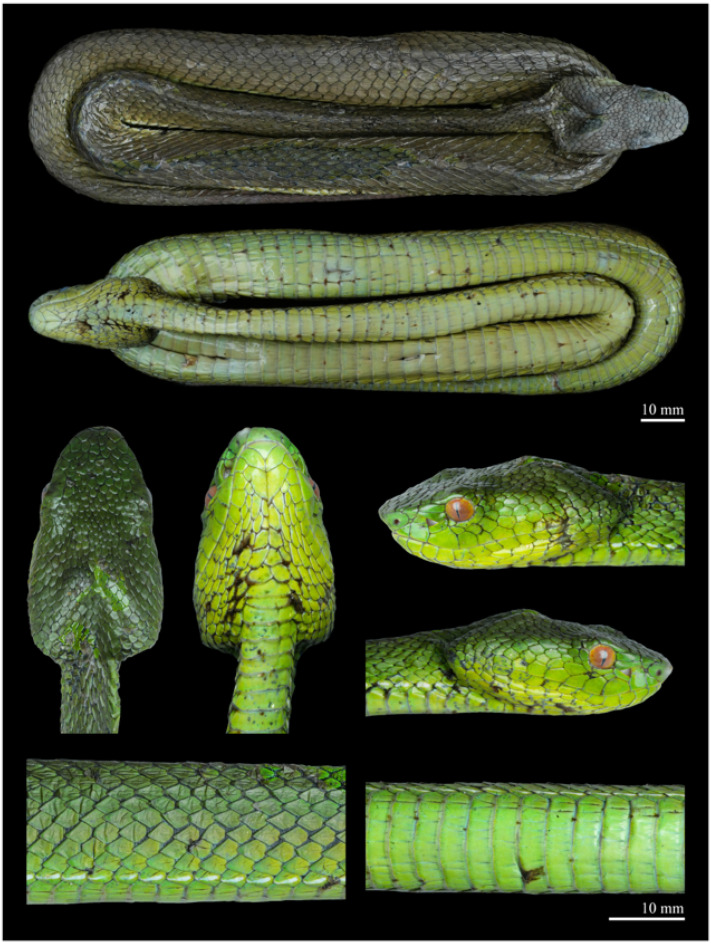
Fresh specimen of the female paratype of *Trimeresurus pretiosus* sp. nov. (QHU R2025021). Photographs by Y.H. Xu. Scale bar = 10 mm.

Variation (Figure 7 and Figure 8). The main morphological characters of *Trimeresurus pretiosus* sp. nov. are summarised in Table 4. The longest known male (QHU R2025020) measures 633 mm TL (SVL 516 mm, TAL 117 mm); and the only known female (QHU R2025021) measures 620 mm TL (SVL 512 mm, TAL 108 mm). The TAL/TL ratio is 0.19 in males and 0.17 in females.

Body scalation. DSR 19–19–15 in all specimens. At midbody, all dorsal scale rows except the outermost are weakly keeled, with keels faint laterally and becoming more distinct medially. The outermost row is completely smooth. VS 140–143 in males and 142 in the female; SC 56–58 in males and 54 in the female, mostly paired with a few single ones interspersed. In the male holotype QHU R2025019, the 3rd, 4th, 57th, and 58th subcaudals are single, while all others are paired; in the male paratype QHU R2025020, the 4th, 5th, 55th, and 56th are single, with the remaining subcaudals paired; and in the female paratype QHU R2025021, the 3rd to 5th, 13th to 16th, 22nd, 24th, and 54th are single, all others paired.

Pattern and colouration (Figure 6(A2,A3,B2,B3,C2,C3), Figure 7 and Figure 8). The species exhibits marked sexual dichromatism. In males, the iris is reddish-brown; the postocular streak is absent (QHU R2025019) or represented by a barely discernible faint whitish streak (QHU R2025020). A broad, distinct, bicoloured ventrolateral stripe runs along the first dorsal scale row. In the female, the iris is orange-yellow, the postocular streak is absent, and the ventrolateral stripe is narrow, white, and restricted to the middle portion of the first dorsal scale row.

**Figure 9:**
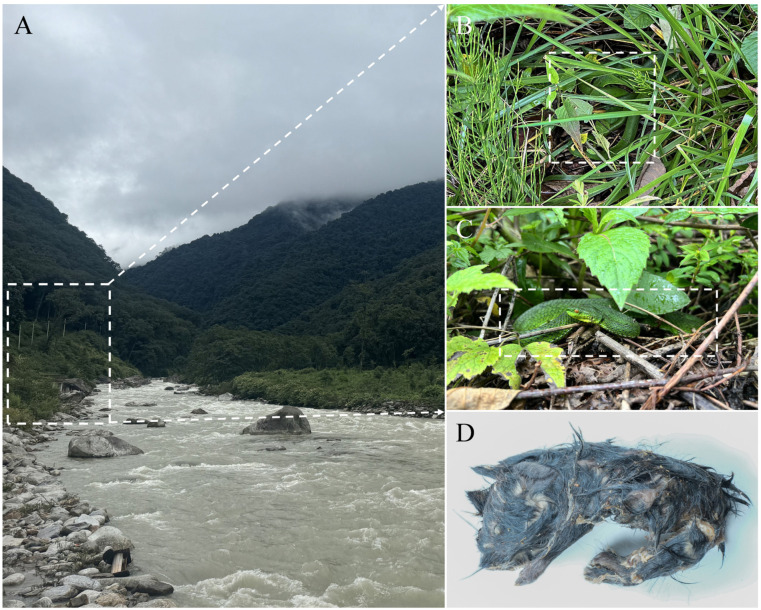
Habitat and field observations of *Trimeresurus pretiosus* sp. nov. (**A**), macrohabitat of the new species in Xiayadong, Yadong, Xizang, China; (**B**), holotype QHU R2025019 in life, in situ; (**C**), paratype QHU R2025021 in life, in situ; (**D**), regurgitated mouse from paratype QHU R2025021. Photographs by Y.H. Xu (**A**,**D**) and Z.Q. Wang (**B**,**C**).

Distribution and natural history. Currently, *Trimeresurus pretiosus* sp. nov. is known only from Xiayadong Township, Yadong County, Xizang AR, China, where it inhabits moist broadleaf forests at an elevation of approximately 1824 m a.s.l. (Figure 9A). All specimens were encountered in the afternoon under clear to partly cloudy conditions. Field observations suggest that the species is predominantly terrestrial, as individuals were found either within grassy vegetation or near rocks surrounded by dense plant cover (Figure 9B,C), often in areas close to riverbanks. We speculate that the riverine environment provides higher humidity and relatively lush vegetation, which may offer suitable microhabitat conditions and sufficient shelter for the species. The female paratype (QHU R2025021) regurgitated a partially digested mouse shortly after capture, indicating that the new species, particularly females, may primarily feed on small mammals including rodents in the wild (Figure 9D). Given its proximity to the borders of Bhutan and India, it is plausible that the new species also occurs in suitable habitats across these regions, a possibility that warrants further field surveys.

**Figure 10:**
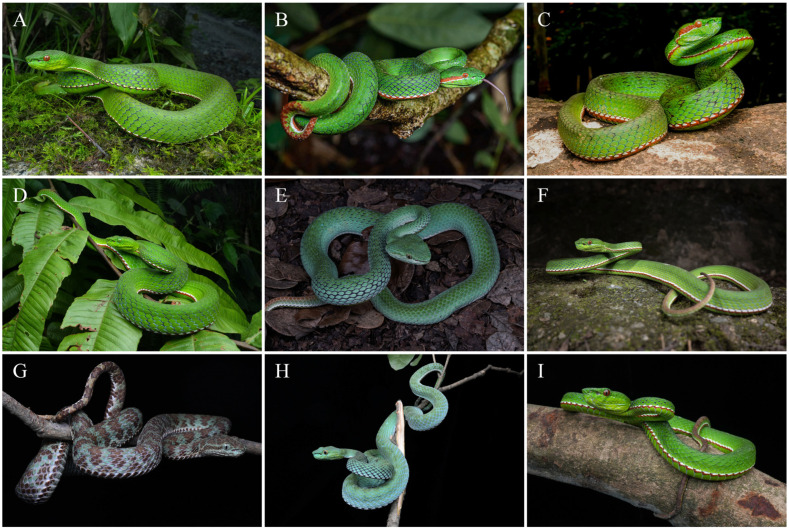
Comparison of males of species in the subgenus *Viridovipera* in life. (**A**), *Trimeresurus pretiosus* sp. nov., unvouchered individual from Xiayadong, Yadong, Xizang, China; (**B**), *T. gumprechti* from Phu Hin Rong Kla NP, Phitsanulok, Thailand; (**C**), *T. mayaae* from Garbhanga Reserve Forest, Guwahati, Assam, India; (**D**), *T. medoensis* from Motuo (Medog), Xizang, China; (**E**), *Trimeresurus* sp. from Fugong, Yunnan, China; (**F**), *T. stejnegeri* from Mt. Wuyi, Fujian, China; (**G**), *T. truongsonensis* from Phong Nha-Ke Bang NP, Quang Binh, Vietnam; (**H**), *T. vogeli* from Phong Nha-Ke Bang NP, Quang Binh, Vietnam; (**I**), *T. yunnanensis* from Tengchong, Yunnan, China. Photographs by Y.H. Xu (**A**,**D**,**G**–**I**), R. Jaihan (**B**), S. Bohra (**C**), T.R. Zhang (**E**), and Z.Q. Wang (**F**).

**Figure 11:**
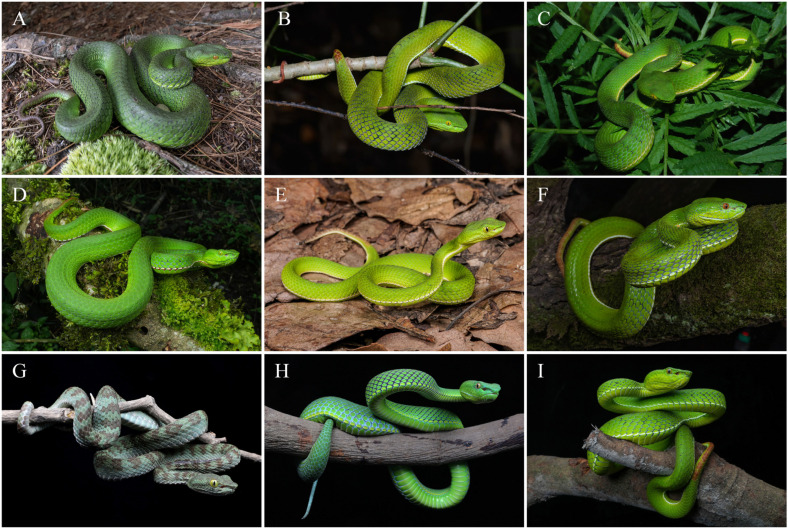
Comparison of females of species in the subgenus *Viridovipera* in life. (**A**). *Trimeresurus pretiosus* sp. nov., unvouchered individual from Xiayadong, Yadong, Xizang, China; (**B**). *T. gumprechti* from Phu Kradueng, Loei, Thailand; (**C**). *T. mayaae* from Shillong, Meghalaya, India; (**D**). *T. medoensis* from Motuo (Medog), Xizang, China; (**E**). *Trimeresurus* sp. from Gongshan, Yunnan, China; (**F**). *T. stejnegeri* from Sanming, Fujian, China; (**G**). *T. truongsonensis* from Phong Nha-Ke Bang NP, Quang Binh, Vietnam; (**H**). *T. vogeli* from Phong Nha-Ke Bang NP, Quang Binh, Vietnam; (**I**). *T. yunnanensis* from Tengchong, Yunnan, China. Photographs by Z.Q. Wang (**A**), R. Jaihan (**B**), G. Vogel (**C**), Y.H. Xu (**D**,**F**,**G**–**I**), and L.F. Peng (**E**).

Comparisons. *Trimeresurus pretiosus* sp. nov. is assigned to the subgenus *Viridovipera* based on its phylogenetic placement as well as on a combination of characteristics, such as the condition of the first supralabial scale (completely separated from the nasal scale) and the morphology of the hemipenis, which is short and spinose, a feature diagnostic of *Viridovipera* [11,14,24]. Accordingly, the morphological comparisons below focus on its congeners within the subgenus *Viridovipera*, which currently comprises seven recognised species and is regarded as the most relevant taxa for differential diagnosis. The principal characteristics distinguishing *Trimeresurus pretiosus* sp. nov. from these species are summarised in Table 5 and illustrated in Figure 10 and Figure 11. Comparative morphological data were compiled from the literature, including Maki [44], Pope [45], Zhao et al. [46], David et al. [47,48,49], Ao et al. [50], Orlov et al. [51], David and Mathew [52], Guo et al. [53], Teynié and David [54], Nguyen et al. [55], Che et al. [15], Rathee et al. [11], Elangbam et al. [24], and Nguyen et al. [14].

*Trimeresurus pretiosus* sp. nov. differs from *T. gumprechti* (distributed in southwestern Yunnan Province [China], Shan State [Myanmar], northern Laos, northern and central Thailand, and northern to central Vietnam) in the following combination of characteristics: smaller maximum SVL in both sexes (516 mm in males, 512 mm in the only examined female vs. 610 mm in males, 654 mm in females); lower number of ventral scales in both sexes (VS 140–143 [mean 141.5 ± 2.1] in males, 142 in the only examined female vs. 156–168 [mean 161.4 ± 3.5] in males, 162–170 [mean 165.7 ± 4.0] in females); lower combined ventral and subcaudal counts in both sexes (VS + SC 198–199 [mean 198.5 ± 0.7] in males, 196 in the only examined female vs. 216–235 [mean 228.3 ± 6.2] in males, 224–234 [mean 229.0 ± 7.1] in females); and subcaudals partly single vs. all paired. It also has fewer dorsal scale rows both anteriorly (ASR 19 vs. 21, rarely 23) and at midbody (MSR 19 vs. 21). Males lack a prominent postocular streak or display only a faint white one, whereas *T. gumprechti* bears a broad red and white stripe behind the eye.

*Trimeresurus pretiosus* sp. nov. differs from *T. mayaae* (distributed across the Indian states of Manipur, Meghalaya, Mizoram, Assam, Nagaland, West Bengal, and Sikkim and the Chin State of northwestern Myanmar) by the following combination of characters: smaller maximum SVL in both sexes (516 mm in males, 512 mm in the only examined female vs. 610 mm in males, 590 mm in females); lower number of ventral scales in both sexes (VS 140–143 [mean 141.5 ± 2.1] in males, 142 in the only examined female vs. 153–163 [mean 158.1 ± 3.0] in males, 152–153 [mean 152.7 ± 0.6] in females); lower combined ventral and subcaudal counts in both sexes (VS + SC 198–199 [mean 198.5 ± 0.7] in males, 196 in the only examined female vs. 211–231 [mean 218.9 ± 6.1] in males, 205–208 [mean 206.7 ± 1.5] in females); and subcaudals partly single vs. all paired. Females have orange-yellow irises, distinctly different from the greenish eyes of *T. mayaae*.

*Trimeresurus pretiosus* sp. nov. differs from *T. medoensis* (restricted to the Xizang AR of southwestern China, northern Myanmar, and northeastern India) by the following combination of characters: smaller maximum SVL in both sexes (516 mm in males, 512 mm in the only examined female vs. 553 mm in males, 624 mm in females); lower number of subcaudals in females (SC 54 vs. 58–60 [mean 59.0 ± 1.0]); subcaudals partly single vs. all paired; and lower combined ventral and subcaudal counts in females (VS + SC 196 vs. 204–206 [mean 205.0 ± 1.0]). It also exhibits more dorsal scale rows anteriorly (ASR 19 vs. 17, rarely 19), midbody (MSR 19 vs. 17), and posteriorly (PSR 15 vs. 13, rarely 11). Eye colouration differs as well: males have reddish-brown irises, females orange-yellow, whereas both sexes of *T. medoensis* have green or yellowish-green eyes. The ventrolateral stripe in females is uniformly white, in contrast to the red and white stripe observed in *T. medoensis*.

*Trimeresurus pretiosus* sp. nov. differs from *T. stejnegeri* (restricted to eastern, southern and southwestern China, including Taiwan Province, northern Vietnam, and northeastern Laos) in the following combination of characteristics: smaller maximum SVL in both sexes (516 mm in males, 512 mm in female vs. 635 mm in males, 670 mm in females); lower number of ventral scales in both sexes (VS 140–143 [mean 141.5 ± 2.1] in males, 142 in the only examined female vs. 154–178 [mean 164.5 ± 4.8] in males, 155–173 [mean 163.9 ± 3.5] in females); lower number of subcaudals in both sexes (SC 56–58 [mean 57.0 ± 1.4] in males, 54 in the only examined female vs. 60–80 [mean 70.3 ± 4.2] in males, 58–60 [mean 59.0 ± 1.0] in females); subcaudals partly single vs. all paired; and lower combined ventral and subcaudal counts in females (VS + SC 198–199 [mean 198.5 ± 0.7] in males, 196 in the only examined female vs. 218–256 [mean 234.9 ± 7.7] in males, 218–237 [mean 227.5 ± 4.5] in females). It further differs in having fewer dorsal scale rows anteriorly (ASR 19 vs. 23, 21 or 25) and at midbody (MSR 19 vs. 21, rarely 23). Males lack a prominent postocular streak or display only a faint white one, while the latter species bears a broad red and white stripe behind the eye. In females, the ventrolateral stripe is uniformly white, in contrast to the white or red and white stripe seen in *T. stejnegeri*.

*Trimeresurus pretiosus* sp. nov. differs from *T. truongsonensis* (restricted to central Vietnam and central Laos) by the following combination of characters: lower number of ventral scales in both sexes (VS 140–143 [mean 141.5 ± 2.1] in males, 142 in the only examined female vs. 170–190 [mean 179.8 ± 8.7] in males, 165–167 [mean 166.0 ± 1.0] in females); lower number of subcaudals in both sexes (SC 56–58 [mean 57.0 ± 1.4] in males, 54 in the only examined female vs. 65–71 [mean 67.4 ± 2.5] in males, 61–70 [mean 66.7 ± 4.9] in females); subcaudals partly single vs. all paired; and lower combined ventral and subcaudal counts in both sexes (VS + SC 198–199 [mean 198.5 ± 0.7] in males, 196 in the only examined female vs. 235–259 [mean 247.2 ± 10.7] in males, 228–235 [mean 232.7 ± 4.0] in females). It further differs in having fewer dorsal scale rows anteriorly (ASR 19 vs. 21) and at midbody (MSR 19 vs. 21). Eye colouration is reddish-brown in males and orange-yellow in females, in contrast to greenish-yellow in both sexes of the latter species. Body colouration is uniformly grass-green, whereas the other species is greenish-blue with broad brown crossbands. The ventrolateral stripe is red and white and wide in males and white in females, compared to red and brown in both sexes. The tail is distinctly reddish-brown dorsally, with this colouration extending from the posterior body to the tail tip, whereas such colouration is absent in *T. truongsonensis*.

*Trimeresurus pretiosus* sp. nov. differs from *T. vogeli* (distributed in the southeastern part of central Thailand, central and southern Laos, Cambodia, and central and southern Vietnam) in the following combination of characteristics: smaller maximum SVL in both sexes (516 mm in males, 512 mm in female vs. 661 mm in males, 947 mm in females); lower number of ventral scales in both sexes (VS 140–143 [mean 141.5 ± 2.1] in males, 142 in the only examined female vs. 157–169 [mean 162.8 ± 3.7] in males, 157–173 [mean 164.2 ± 5.8] in females); lower number of subcaudals in both sexes (SC 56–58 [mean 57.0 ± 1.4] in males, 54 in the only examined female vs. 63–71 [mean 67.0 ± 2.3] in males, 59–70 [mean 63.0 ± 3.8] in females); subcaudals partly single vs. all paired; lower combined ventral and subcaudal counts in both sexes (VS + SC 198–199 [mean 198.5 ± 0.7] in males, 196 in the only examined female vs. 221–238 [mean 229.8 ± 4.9] in males, 218–238 [mean 227.7 ± 6.3] in females). It further differs in having fewer dorsal scale rows anteriorly (ASR 19 vs. 21, occasionally 20 or 23) and at midbody (MSR 19 vs. 21, occasionally 20). Eye colouration in males is reddish-brown (vs. light orange). The ventrolateral stripe is white in females (vs. pale yellow and white, thin). The tail is distinctly reddish-brown dorsally, with this colouration extending from the posterior body to the tail tip, whereas such colouration is absent in *T. vogeli*.

Finally, *Trimeresurus pretiosus* sp. nov. differs from *T. yunnanensis* (restricted to the central Yunnan and southern Sichuan provinces of China and the Mandalay Region of Myanmar) in the following combination of characteristics: smaller maximum SVL in both sexes (516 mm in males, 512 mm in the only examined female vs. 602 mm in males, 804 mm in females); lower number of ventral scales in both sexes (VS 140–143 [mean 141.5 ± 2.1] in males, 142 in the only examined female vs. 151–164 [mean 158.2 ± 3.9] in males, 150–164 [mean 157.9 ± 3.6] in females); lower number of subcaudals in males (SC 56–58 [mean 57.0 ± 1.4] vs. 61–71 [mean 66.1 ± 2.9]); lower combined ventral and subcaudal counts in both sexes (VS + SC 198–199 [mean 198.5 ± 0.7] in males, 196 in the only examined female vs. 221–231 [mean 225.9 ± 3.9] in males, 211–223 [mean 216.4 ± 3.7] in females). Males lack a prominent postocular streak or display only a faint white one, while the latter species bears a broad red and white stripe behind the eye. The ventrolateral stripe in females is uniformly white, in contrast to the thin pale green stripe in *T. yunnanensis*.

## 4. Discussion

The discovery of *Trimeresurus pretiosus* sp. nov. constitutes an important addition to our knowledge on the taxonomy of the subgenus *Viridovipera*, as it represents the westernmost and most geographically isolated member of the group. Mitochondrial DNA genealogy indicates that the new species is a sister to the clade comprising *T. mayaae*, *T. medoensis*, and *T.* cf. *medoensis*. Although the new species is currently known only from Yadong County of Xizang AR of China, its occurrence near the People’s Republic of China’s national borders with Bhutan and India suggests that it may likely also inhabit the adjacent regions, underscoring the need for targeted surveys in these areas.

Given its highly restricted known distribution and apparent rarity, *Trimeresurus pretiosus* sp. nov. may warrant conservation attention. Although detailed population data are lacking, the species is currently known only from a single locality in Yadong County. Its habitat lies within a region subject to increasing anthropogenic pressures, including road construction and potential climate-related habitat shifts. These factors may pose significant threats to its survival. We therefore recommend that *Trimeresurus pretiosus* sp. nov. be provisionally considered as vulnerable (VU) under IUCN criteria, pending further surveys to clarify its population status, distributional range, and habitat requirements.

Our data also provide new information on the intrageneric taxonomy of the subgenus *Viridovipera*. *Trimeresurus gumprechti* was previously considered a widely distributed species, recorded in Thailand, Laos, Vietnam, Myanmar, and China [13,22,27,48]. However, our results suggest that geographically distinct populations within this species do not form a monophyletic group. The specimens from southern Yunnan, previously identified as *T. gumprechti*, form a distinct lineage that exhibit considerable genetic divergence from *T. gumprechti* sensu stricto, with uncorrected *p*-distances in cyt *b* ranging from 4.1% to 6.2% (Figure 2, Table 2). They may likely represent a new undescribed species of the subgenus *Viridovipera*. Furthermore, although mitochondrial DNA sequences of *T. yunnanensis* are available, none have been obtained from topotypic material; the current sequences originate from specimens in Huili City, Sichuan Province, China, which are geographically distant and may be possibly divergent [53]. Confirming the genetic identity of *T. yunnanensis* using material from the type locality should therefore also be a priority.

Despite several decades of herpetological surveys in Yadong County since the 1970s, this is the first confirmed record of a *Trimeresurus* species from the area [15,56]. This discovery highlights both the rarity of the species and the persistent gaps in current biodiversity inventories of Yadong County. Our findings suggest that additional previously overlooked taxa may persist in these montane regions, particularly within the microhabitats below 2000 m a.s.l. along the southern slopes of the Himalayas. Continued fieldwork in these underexplored habitats is essential to enhance our understanding of regional reptile diversity and to inform conservation planning in this biogeographically complex region.

This finding also underscores the need to reassess the snake fauna of the Xizang AR, as species composition has likely shifted since the most recent comprehensive accounts (e.g., Che et al. [15]). In light of recent research advances, we have accordingly updated the checklist of snake species known from the Xizang AR, China (Table 6), raising the total number of recorded species to 67 (based on data from Che et al. [15]; Huang et al. [57]; Shi et al. [58]; David et al. [59]; Guo et al. [60]; Ren et al. [61]; Guo et al. [62]; Guo and Che [63]; Shu et al. [64]; Weng et al. [65]; Bohra et al. [66]; Jiang et al. [67,68]; Nguyen et al. [69]; Patel et al. [70]; Ren et al. [71]).

## 5. Conclusions

We described a new species of green pit viper of the genus *Trimeresurus*, *T. pretiosus* sp. nov., from southern Xizang AR, China, based on morphological and molecular data. The new species represents the currently known westernmost member of the subgenus *Viridovipera*, bringing the total number of recognised species in the subgenus to eight. Its discovery also raises the checklist of snake species recorded from the Xizang AR to 67, underscoring the region’s underestimated herpetofaunal diversity. *Trimeresurus pretiosus* sp. nov. is currently known only from the type locality in Yadong County, where it has not been previously recorded despite several decades of herpetological surveys. Given its restricted distribution, apparent rarity, and exposure to potential anthropogenic threats, we recommend that the species be provisionally classified as Data Deficient under IUCN criteria, pending further studies. Future research should focus on clarifying its distributional range, habitat preferences, population size, and phylogenetic position within the *T. medoensis* species complex. Continued surveys in Yadong and adjacent regions of Bhutan and India are essential, both to confirm its broader range and to strengthen conservation planning for this biogeographically significant sector of the Himalayas. Ultimately, this discovery highlights the Himalayan region as a global hotspot for pit viper diversity and endemism, emphasising its importance for future taxonomic, biogeographic, and conservation studies.

## Figures and Tables

**Table 1:** GenBank accession numbers, localities, and voucher information for all specimens used in this study.

NO.	Species Name	Locality	Voucher NO.	*16S*	cyt *b*	*ND4*	References
**Genus *Trimeresurus***							
**Subgenus *Viridovipera***							
1	*Trimeresurus pretiosus* sp. nov.	Yadong, Xizang, China	QHU R2025019	PX061894	PX068368	PX094011	This study
2	*Trimeresurus pretiosus* sp. nov.	Yadong, Xizang, China	QHU R2025020	PX061895	PX068369	PX094010	This study
3	*Trimeresurus pretiosus* sp. nov.	Yadong, Xizang, China	QHU R2025021	PX061895	PX068370	PX094012	This study
4	*T. gumprechti*	Loei, Thailand	AM A164	AF517181	AY352766	AF517224	Malhotra and Thorpe [13]; Creer et al. [21]
5	*T. gumprechti*	Pu Mat NP, Nghe An, Vietnam	AM B174	AY059563	AY059573	AY059595,	Malhotra and Thorpe [22]
6	*T. gumprechti*	Jingdong, Yunnan, China	GP 851	–	KT216392	KT216441	Guo et al. [23]
7	*T. gumprechti*	Lincang, Yunnan, China	GP 3507	KT216339	KT216384	KT216433	Guo et al. [23]
8	*T. gumprechti*	Lincang, Yunnan, China	GP 3509	KT216341	KT216386	KT216435	Guo et al. [23]
9	*T. gumprechti*	Longling, Yunnan, China	KIZ 05089	KT216352	KT216399	KT216448	Guo et al. [23]
10	*T. gumprechti*	Jingdong, Yunnan, China	KIZ 047083	KT216351	KT216398	KT216447	Guo et al. [23]
11	*T.* cf. *gumprechti*	Honghe, Yunnan, China	GP 2532	KT216331	KT216376	KT216425	Guo et al. [23]
12	*T.* cf. *gumprechti*	Pingbian, Yunnan, China	GP 3273	KT216334	KT216379	KT216428	Guo et al. [23]
13	*T.* cf. *gumprechti*	Pingbian, Yunnan, China	GP 3275	KT216336	KT216381	KT216430	Guo et al. [23]
14	*T.* cf. *gumprechti*	Mengzi, Yunnan, China	GP 3564	KT216344	KT216389	KT216438	Guo et al. [23]
15	*T.* cf. *gumprechti*	Mengzi, Yunnan, China	GP 3589	KT216345	KT216390	KT216439	Guo et al. [23]
16	*T. mayaae*	Champhai, Mizoram, India	NCBS NRC-AA-0012	–	OM966859	–	Rathee et al. [11]
17	*T. mayaae*	Ri-Bhoi, Meghalaya, India	BNHS 3658	–	OM966860	–	Rathee et al. [11]
18	*T. mayaae*	Ri-Bhoi, Meghalaya, India	VR/ERS/ZSI/833	–	OM966862	–	Rathee et al. [11]
19	*T. mayaae*	Kangpokpi, Manipur, India	MZMU 2970	–	OQ968476	–	Elangbam et al. [24]
20	*T. mayaae*	Kangpokpi, Manipur, India	MZMU 2971	PP566116	–	–	Elangbam et al. [24]
21	*T. medoensis*	Motuo (Medog), Xizang, China	SYS r001831/CHS 824	MK194252	MK201553	–	Li et al. [25]
22	*T. medoensis*	Motuo (Medog), Xizang, China	KIZ YPX46122	MW020095	MW111479	–	Che et al. [15]
23	*T. medoensis*	Motuo (Medog), Xizang, China	KIZ YPX46123	MW020331	MW133479	–	Che et al. [15]
24	*T. medoensis*	Motuo (Medog), Xizang, China	ANU ZR24025	–	PX068371	PX094008	This study
25	*T. medoensis*	Motuo (Medog), Xizang, China	ANU ZR24026	–	PX068372	PX094007	This study
26	*T. medoensis*	Motuo, Xizang, China	ANU ZR24072	–	PX068373	–	This study
27	*T.* cf. *medoensis*	Kachin, Myanmar	AM B416/CAS 221528	AY352735	AY352765	AY352831	Malhotra and Thorpe [13]
28	*T.* cf. *medoensis*	Northeast India	V18	MG995794	MG995819	MG995834	Unpublished
29	*Trimeresurus* sp.	Fugong, Yunnan, China	ANU ZR24133	PX061893	PX068374	PX094009	This study
30	*T. stejnegeri*	Mt. Wuyi, Fujian, China	GP 816	–	KX019140	KX019294	Guo et al. [26]
31	*T. stejnegeri*	Mt. Jinggang, Jiangxi, China	GP 662	–	KX019120	KX019274	Guo et al. [26]
32	*T. stejnegeri*	Qimen, Anhui, China	GP 470	–	KX019094	KX019248	Guo et al. [26]
33	*T. stejnegeri*	Jinhua, Zhejiang, China	GP 633	–	KX019111	KX019265	Guo et al. [26]
34	*T. stejnegeri*	Youxi, Fujian, China	GP 755	–	KX019131	KX019285	Guo et al. [26]
35	*T. stejnegeri*	Dehua, Fujian, China	GP 2435	–	KX019054	KX019209	Guo et al. [26]
36	*T. stejnegeri*	Taichung, Taiwan, China	AM TST23	–	AF277689	EU443799	Creer et al. [21]; Dawson et al. [27]
37	*T. stejnegeri*	Mt. Diaoluo, Hainan, China	GP 45	–	KX019088	KX019242	Guo et al. [26]
38	*T. stejnegeri*	Qiongzhong, Hainan, China	GP 46	–	KX019089	KX019243	Guo et al. [26]
39	*T. stejnegeri*	Qiongzhong, Hainan, China	GP 48	–	KX019104	KX019258	Guo et al. [26]
40	*T. stejnegeri*	Cat Ba NP, Hai Phong, Vietnam	GP 881	–	KX019145	KX019299	Guo et al. [26]
41	*T. stejnegeri*	Phia Oac-Phia Den NP, Cao Bang, Vietnam	ROM 35321	–	KT216408	KT216457	Guo et al. [26]
42	*T. stejnegeri*	Nonggang, Guangxi, China	GP 2461	–	KX019056	KX019211	Guo et al. [26]
43	*T. stejnegeri*	Nanchuan, Chongqing, China	GP 1227	–	KX019007	KX019162	Guo et al. [26]
44	*T. stejnegeri*	Leishan, Guizhou, China	GP 1887	–	KX019034	KX019189	Guo et al. [26]
45	*T. stejnegeri*	Luoding, Guangdong, China	KIZ 09939	–	KX019158	KX019312	Guo et al. [26]
46	*T. stejnegeri*	Enshi, Hubei, China	GP 2011	–	KX019040	KX019195	Guo et al. [26]
47	*T. truongsonensis*	Phong Nha-Ke Bang NP, Quang Binh, Vietnam	AM B659/VNUH 190606	EU443818	EU443815	EU443816	Dawson et al. [27]
48	*T. truongsonensis*	Khammouane, Laos	ZMMU NAP-09115	–	PX068365	PX094015	This study
49	*T. vogeli*	Nakhon Ratchasima, Thailand	AM B97	AY059562	AY059574	AY059596	Malhotra and Thorpe [13]
50	*T. vogeli*	Nakhon Ratchasima, Thailand	ZMMU NAP-08227	–	PX068366	PX094013	This study
51	*T. vogeli*	Nakhon Ratchasima, Thailand	ZMMU NAP-08228	–	PX068367	PX094014	This study
52	*T. yunnanensis*	Huili, Sichuan, China	GP 37	EU443812	EF597522	EF597527	Dawson et al. [27]
53	*T. yunnanensis*	Huili, Sichuan, China	GP 38	EU443814	EF597523	EF597528	Dawson et al. [27]
**Subgenus *Himalayophis***							
54	*T. arunachalensis*	Northeast India	APF/SFRI-1871	MK722155	MK720609	–	Captain et al. [28]
55	*T. tibetanus*	Nepal	AM B258	AY352715	AY352749	AY352810	Malhotra and Thorpe [13]
**Subgenus *Parias***							
56	*T. flavomaculatus*	Mindanao, Philippines	AM B4	AY352734	AY352764	AY352830	Malhotra and Thorpe [13]
57	*T. hageni*	Songhkla, Thailand	AM B33	AY059552	AY059567	AY059585	
58	*T. malcolmi*	Mt. Kinabalu, Sabah, Borneo, Malaysia	AM B349	AY371786	AY371832	AY371861	Malhotra and Thorpe [13]
59	*T. mcgregori*	Bataan islands, Philippines	AM B289	AY371795	AY371831	AY371858	Malhotra and Thorpe [13]
60	*T. schultzei*	Palawan, Philippines	AM B210	AY352725	AY352756	AY352819	Malhotra and Thorpe [13]
61	*T. sumatranus*	Bengkulu, Sumatra, Indonesia	AM B367	AY371791	AY371824	AY371864	Malhotra and Thorpe [13]
**Subgenus *Sinovipera***							
62	*T. sichuanensis*	Sichuan, China	GP7/YBU030116	HQ850449	HQ850447	HQ850446	Guo and Wang [29]
**Subgenus *Popeia***							
63	*T. lanna*	Doi Inthanon NP, Chiangmai, Thailand	AUP-00061	OR471637	OR470571	OR470534	Idiiatullina et al. [4]
64	*T. nebularis*	Cameron Highlands, Pahang, Malaysia	AM B345	AY371775	AY371811	AY371849	Sanders et al. [30]
65	*T. phuketensis*	Phang Nga, Thailand	AM B467	AY371781	AY371807	AY371851	Sanders et al. [30]
66	*T. popeiorum*	Yingjiang, Yunnan, China	DL2017070101	MH779887	MH779875	MH779879	Chen et al. [31]
67	*T. sabahi sabahi*	Mt. Kinabalu, Sabah, Borneo, Malaysia	AM B344	AY371771	AY371815	AY371842	Malhotra and Thorpe [13]
68	*T. tenasserimensis*	Suan Phueng, Ratchaburi, Thailand	ZMMU Re-17669	PP032802	OR999089	PP032781	Idiiatullina et al. [4]
**Subgenus *Trimeresurus***							
69	*T. albolabris*	Shek Kwu Chan, Hong Kong, China	AM A157	AY352744	AF171884	AY352839	Malhotra and Thorpe [13]; Malhotra and Thorpe [32]
70	*T. cantori*	Nicobar Is., India	AM A85	AY352741	AF171889	AY352836	Malhotra and Thorpe [13]; Malhotra and Thorpe [32]
71	*T. caudornatus*	Yingjiang, Yunnan, China	AR1238	MK575042	MK575036	MK575038	Chen et al. [33]
72	*T. ciliaris*	Thum Khao Ting, Trang, Thailand	ZMMU Re-17661	OR471621	OR470557	OR470538	Idiiatullina et al. [3]
73	*T. erythrochloris*	Tham Si Va Cave, Klong Hat, Sa Kaeo, Thailand	RIM-0079	PQ654052	PQ658816	PQ658818	Pawangkhanant et al. [7]
74	*T. erythrurus*	Rangoon, Myanmar	AM A209	AF517174	AF171900	AF517217	Creer et al. [19]
75	*T. kanburiensis*	Khao Yai NP, Kanchanaburi, Thailand	ZMMU Re-17667	–	OR470579	OR470553	Idiiatullina et al. [3]; Idiiatullina et al. [5]
76	*T. macrops*	Bangkok, Thailand	ZMMU Re-17856	–	PP766219	PP779475	Idiiatullina et al. [6]
**Out group**							
77	*Craspedocephalus puniceus*	Indonesia	AM B213	AF517177	AF517192	AF517220	Creer et al. [19]
78	*Peltopelor malabaricus*	Tamil Nadu, India	AM A218	AY059564	AY059569	AY059587	Malhotra and Thorpe [13]

**Table 2:** Uncorrected *p*-distances (%) between the sequences of cyt *b* gene of species of the subgenus *Viridovipera* included in the phylogenetic analyses. The values represent the minimum and maximum observed genetic divergence between species.

NO.	Species	1	2	3	4	5	6	7	8	9	10	11
1	*T. pretiosus* sp. nov.	0.2–0.5										
2	*T. gumprechti*	9.0–10.8	0.0–3.5									
3	*T.* cf. *gumprechti*	8.1–9.5	4.1–6.2	0.0–0.4								
4	*T. mayaae*	6.0–7.1	9.5–12.1	8.2–10.2	0.0–1.5							
5	*T.* cf. *medoensis*	5.8–6.3	9.2–11.0	7.5–8.9	4.4–5.1	2.2						
6	*T. medoensis*	6.6–7.5	10.0–12.5	10.1–11.6	5.1–5.9	6.7–6.9	0.0					
7	*Trimeresurus* sp.	7.9–8.2	5.6–7.6	5.6–6.1	8.3–8.5	7.2–7.7	8.3–9.6	–				
8	*T. stejnegeri*	8.5–12.5	5.1–8.1	4.4–6.9	8.2–12.1	8.1–12.5	8.2–12.1	6.4–8.1	0.1–7.9			
9	*T. truongsonensis*	9.3–10.6	6.9–10.5	8.0–9.4	8.8–9.4	8.5–9.0	9.3–10.7	7.6–8.5	7.6–9.9	3.3		
10	*T. vogeli*	10.6–11.9	9.3–12.8	8.7–11.5	7.1–10.2	8.8–9.9	9.2–11.1	8.3–9.3	9.6–13.3	9.2–10.9	0.1–0.2	
11	*T. yunnanensis*	7.7–7.8	9.0–10.6	9.4–9.5	7.1–7.5	7.1–7.3	8.4–9.1	7.7–7.8	8.0–11.7	7.5–9.3	9.1–9.8	0.1

**Table 3:** Uncorrected *p*-distances (%) between the sequences of *ND4* gene of species of the subgenus *Viridovipera* included in the phylogenetic analyses. The values represent the minimum and maximum observed genetic divergence between species.

NO.	Species	1	2	3	4	5	6	7	8	9	10
1	*T. pretiosus* sp. nov.	0.0									
2	*T. gumprechti*	8.3–9.4	0.0–1.4								
3	*T.* cf. *gumprechti*	8.6–9.3	2.9–4.7	0.0–0.2							
4	*T. medoensis*	7.0	8.0–9.9	7.1–8.7	0.0						
5	*T.* cf. *medoensis*	7.8–7.9	8.9–10.1	7.9–9.6	6.5–6.8	0.0					
6	*Trimeresurus* sp.	8.2	4.9–5.4	5.4–6.6	9.4	8.8–9.1	–				
7	*T. stejnegeri*	7.8–9.5	3.1–5.7	3.2–6.3	8.1–9.3	7.6–10.5	5.5–7.6	0.0–6.2			
8	*T. truongsonensis*	9.1–10.0	7.3–8.7	6.8–8.3	9.3–10.3	8.5–8.9	7.4–8.3	6.7–9.7	2.2		
9	*T. vogeli*	10.1–11.0	7.6–8.7	8.1–9.7	9.5–10.3	11.4–12.1	8.2–8.4	7.1–9.6	7.2–8.7	0.0–0.2	
10	*T. yunnanensis*	6.7–7.4	6.0–7.2	6.2–6.8	7.4–8.6	7.0–7.4	6.6–7.0	5.8–8.8	6.8–7.4	7.9–8.2	0.2

**Table 4:** Main measurements (in mm) and meristic characters of the type series of *Trimeresurus pretiosus* sp. nov.

Specimen Voucher	QHU R2025019	QHU R2025020	QHU R2025021
Type	Holotype	Paratype	Paratype
Sex	Male	Male	Famale
SVL (mm)	451	516	512
TAL (mm)	108	117	108
TL (mm)	559	633	620
HL (mm)	24.2	24.9	28.2
HW (mm)	16.1	17.6	17.4
ESD (mm)	6.7	6.5	7.2
EN (mm)	5.8	5.6	5.9
ED (mm)	3.8	3.5	3.8
Internasals	significantly enlarged	significantly enlarged	slightly enlarged
INS	1	1	2
PRO	3/3	3/3	3/3
PO	2/3	1/1	2/2
SO	1	1	1
SOL (mm)	4.7	5.2	5.6
SOW (mm)	2.1	2.0	2.2
SBO	1	1	1
IOS	9	11	10
SpOC	8/7	8/7	8/7
TEMP-keeled	slightly keeled	slightly keeled	almost smooth
SL	9/9	8/8	9/9
IL	11/10	10/10	11/11
DSR	19–19–15	19–19–15	19–19–15
VS	140	143	142
SC	58	56	54
SC single or paired	the 3rd, 4th, 57th, and 58th are single, all others paired	the 4th, 5th, 55th, and 56th are single, all others paired	the 3rd to 5th, 13th–16th, 22nd, 24th, and 54th are single, all others paired
VS + SC	198	199	196
Eye colour	reddish-brown	reddish-brown	orange-yellow
Postocular streak	absent	very faint, thin white	absent
Ventrolateral stripe	red + white, wide	red + white, wide	white, thin

**Table 5:** Summary of morphological characters in members of the subgenus *Viridovipera*.

*Trimeresurus*	Max SVL (mm)		VS		SC		VS + SC		SC Single or Paired	ASR	MSR	PSR	SL	IL	Eye Colour		Postocular Streak		Ventrolateral Stripe		Tail Red	Body Colouration	Sources
	♂	♀	♂	♀	♂	♀	♂	♀							♂	♀	♂	♀	♂	♀			
*T. pretiosus* sp. nov.	516	512	140–143 [141.5 ± 2.1]	142	56–58 [57.0 ± 1.4]	54	198–199 [198.5 ± 0.7]	196	partly single	19	19	15	9 (8)	10 or 11	reddish-brown	orange-yellow	none or very faint, thin white	none	red + white, wide	white, thin	yes	uniformly grass-green	(17)
*T*. *gumprechti*	610	654	156–168 [161.4 ± 3.5]	162–170 [165.7 ± 4.0]	56–73 [66.4 ± 5.6]	54–69 [61.5 ± 10.6]	216–235 [228.3 ± 6.2]	224–234 [229.0 ± 7.1]	all paired	21 (23)	21	15	10 (9, 11)	12 (11, 13, 14)	bright or deep red	golden yellow	red + white	none or faint white	red + white, wide	white	yes	uniformly grass-green	(3), (6), (16)
*T*. *mayaae*	610	590	153–163 [158.1 ± 3.0]	152–153 [152.7 ± 0.6]	54–69 [61.1 ± 4.5]	53–55 [54.0 ± 1.0]	211–231 [218.9 ± 6.1]	205–208 [206.7 ± 1.5]	all paired	21 (19, 23, 25, 28)	21 (19, 20)	15 (16, 17)	9 (10, 8)	11 (10, 12, 13)	rusty or greenish	greenish	none or red + white	none	red + white, wide	pale yellow + white, thin	yes	uniformly grass-green	(7), (8), (14), (15), (16)
*T*. *medoensis*	553	624	146–151 [148.6 ± 1.8]	145–147 [146.0 ± 1.0]	55–59 [57.0 ± 1.4]	58–60 [59.0 ± 1.0]	201–208 [205.6 ± 2.9]	204–206 [205.0 ± 1.0]	all paired	17 (19)	17	13 (11)	8 (9)	10 (8, 9)	green or yellowish green	green or yellowish green	none or faint white	none	red + white, wide	red + white	yes	uniformly grass-green	(4), (13), (16)
*T*. *stejnegeri*	635	670	154–178 [164.5 ± 4.8]	155–173 [163.9 ± 3.5]	60–80 [70.3 ± 4.2]	58–70 [63.4 ± 2.9]	218–256 [234.9 ± 7.7]	218–237 [227.5 ± 4.5]	all paired	21 (23, 25)	21 (23)	15	10 (9, 11, 12)	11 (10, 12, 13, 14)	bright red or amber (rarely yellow)	yellow or amber	red + white	none (or very faint)	red + white, wide	red + white or white	yes	uniformly grass-green	(1), (3), (16)
*T*. *truongsonensis*	521	488	170–190 [179.8 ± 8.7]	165–167 [166.0 ± 1.0]	65–71 [67.4 ± 2.5]	61–70 [66.7 ± 4.9]	235–259 [247.2 ± 10.7]	228–235 [232.7 ± 4.0]	all paired	21	21	15	10 (9, 11)	12 (11, 13)	greenish-yellow	greenish-yellow	none	none	red + brown, wide	red + brown	no	greenish blue with brown broad bands	(8), (11), (16)
*T*. *vogeli*	661	947	157–169 [162.8 ± 3.7]	157–173 [164.2 ± 5.8]	63–71 [67.0 ± 2.3]	59–70 [63.0 ± 3.8]	221–238 [229.8 ± 4.9]	218–238 [227.7 ± 6.3]	all paired	21 (20, 23)	21 (20)	15	11 (9, 10)	12 (11, 13, 14, 15, 16)	light orange	light orange	none or faint white	none	red + white, wide	pale yellow + white, thin	no	uniformly grass-green	(5), (16)
*T*. *yunnanensis*	602	804	151–164 [158.2 ± 3.9]	150–164 [157.9 ± 3.6]	61–71 [66.1 ± 2.9]	52–65 [57.8 ± 3.6]	221–231 [225.9 ± 3.9]	211–223 [216.4 ± 3.7]	all paired	19 (20, 21)	19 (20)	15 (17)	10 (9, 11)	12 (10, 11, 13)	bright or deep red	golden yellow	red + white	none	red + white, wide	pale white, thin	yes	uniformly grass-green	(2), (3), (10), (16)

Sources: (1) = Maki [44]; (2) = Pope [45]; (3) = Zhao et al. [46]; (4) = David et al. [47]; (5) = David et al. [48]; (6) = David et al. [49]; (7) = Ao et al. [50]; (8) = Orlov et al. [51]; (9) = David and Mathew [52]; (10) = Guo et al. [53]; (11) = Teynié and David [54]; (12) = Nguyen et al. [55]; (13) = Che et al. [15]; (14) = Rathee et al. [11]; (15) = Elangbam et al. [24]; (16) = Nguyen et al. [14]; (17) = this study.

**Table 6:** Updated list of snakes (Serpentes) from Xizang AR, China.

NO.	Scientific Name
**Colubridae**	
1	*Ahaetulla flavescens* (Wall, 1910)
2	*Anguiculus rappii* (Günther, 1860)
3	*Boiga gocool* (Gray, 1834)
4	*Boiga stoliczkae* Wall, 1909
5	*Dendrelaphis biloreatus* Wall, 1908
6	*Dendrelaphis* cf. *cyanochloris* (Wall, 1921)
7	*Elaphe cantoris* (Boulenger, 1894)
8	*Elaphe carinata* (Günther, 1864)
9	*Elaphe hodgsonii* (Günther, 1860)
10	*Elaphe taeniura* Cope, 1861
11	*Euprepiophis mandarinus* (Cantor, 1842)
12	*Gonyosoma prasinum* (Blyth, 1854)
13	*Oreocryptophis porphyraceus* (Cantor, 1839)
14	*Liopeltis frenatus* (Günther, 1858)
15	*Lycodon latifasciatus* Nguyen, Lee, Jiang, Ding, Chit, Poyarkov and Vogel, 2025
16	*Lycodon gammiei* (Blanford, 1878)
17	*Lycodon gongshan* Vogel and Luo, 2011
18	*Lycodon jara* (Shaw, 1802)
19	*Lycodon septentrionalis* (Günther, 1875)
20	*Lycodon zayuensis* Jiang, Wang, Jin and Che, 2020
21	*Oligodon albocinctus* (Cantor, 1839)
22	*Oligodon juglandifer* (Wall, 1909)
23	*Oligodon melanozonatus* Wall, 1922
24	*Oligodon zhangfujii* Jiang, Wu, Huang, Ren, Gao, Lyu and Li, 2024
26	*Platyceps rhodorachis* (Jan, 1863)
27	*Ptyas nigromarginata* (Blyth, 1854)
**Dipsadidae**	
28	*Thermophis baileyi* (Wall, 1907)
**Natricidae**	
29	*Hebius lacrima* Purkayastha and David, 2019
30	*Hebius* cf. *khasiensis* (Boulenger, 1890)
31	*Herpetoreas burbrinki* Guo, Zhu, Liu, Zhang, Li, Huang and Pyron, 2014
32	*Herpetoreas platyceps* (Blyth, 1854)
33	*Herpetoreas* cf. *sieboldii* Günther, 1860
34	*Herpetoreas tpser* Ren, Jiang, Huang, David and Li, 2022
35	*Rhabdophis himalayanus* (Günther, 1864)
36	*Rhabdophis leonardi* (Wall, 1923)
37	*Smithophis arunachalensis* Das, Deepak, Captain, Wade and Gower, 2020
38	*Trachischium apteii* Bhosale, Gowande and Mirza, 2019
39	*Trachischium fuscum* (Blyth, 1855)
40	*Trachischium monticola* (Cantor, 1839)
41	*Trachischium nyalamense* Guo, Liu, Jin, Shu, Wu and Che, 2024
42	*Trachischium* cf. *laeve* Peracca, 1904
43	*Trachischium reticulatum* (Blyth, 1855)
44	*Trachischium tenuiceps* (Blyth, 1854)
**Paretidae**	
45	*Pareas monticola* (Cantor, 1839)
**Pseudoxenodontidae**	
46	*Pseudoxenodon macrops* (Blyth, 1854)
**Psammodynastidae**	
47	*Psammodynastes pulverulentus* (Boie, 1827)
**Sibynophiidae**	
48	*Sibynophis collaris* (Gray, 1853)
**Elapidae**	
49	*Bungarus bungaroides* (Cantor, 1839)
50	*Bungarus lividus* Cantor, 1839
51	*Bungarus niger* Wall, 1908
52	*Naja kaouthia* Lesson, 1831
53	*Ophiophagus hannah* (Cantor, 1836)
54	*Sinomicrurus macclellandi* (Reinhardt, 1844)
**Viperidae**	
55	*Gloydius lipipengi* Shi, Liu and Malhotra, 2021
56	*Gloydius huangi* Wang, Ren, Dong, Jiang, Siler and Che, 2019
57	*Gloydius variegatus* Ren, Huang, Wu, Jiang and Li, 2024
58	*Ovophis monticola* (Günther, 1864)
59	*Ovophis zayuensis* (Jiang, 1977)
60	*Protobothrops himalayanus* Pan, Chettri, Yang, Jiang, Wang, Zhang and Vogel, 2013
61	*Protobothrops jerdonii* (Günther, 1875)
62	*Protobothrops kaulbacki* (Smith, 1940)
63	*Trimeresurus* (*Viridovipera*) *medoensis* Djao, 1977
64	*Trimeresurus* (*Viridovipera*) *pretiosus* sp. nov.
65	*Trimeresurus* (*Himalayophis*) *arunachalensis* Captain, Deepak, Pandit, Bhatt and Athreya, 2019
66	*Trimeresurus* (*Himalayophis*) *tibetanus* Huang, 1982
67	*Trimeresurus* (*Trimeresurus*) *salazar* Mirza, Bhosale, Phansalkar, Sawant, Gowande and Patel, 2020

## Data Availability

The data presented in this study are available on request from the corresponding author. ZooBank Code: urn:lsid:zoobank.org:act:9941DC32-7E6F-49AD-AC2D-B286CB0BD96A; urn:lsid:zoobank.org:pub:F4CF8047-E2EE-4AD0-8524-4FDAF63DA40D.

## References

[1] Mirza Z.A., Lalremsanga H.T., Bhosale H., Gowande G., Patel H., Idiiatullina S.S., Poyarkov N.A. (2023). Systematics of *Trimeresurus popeiorum* Smith, 1937 with a revised molecular phylogeny of Asian pit vipers of the genus *Trimeresurus* Lacépède, 1804 sensu lato. Evol. Syst..

[2] Vogel G., Nguyen T.V., David P. (2023). A new green pitviper of the *Trimeresurus albolabris* complex (Reptilia: Serpentes: Viperidae) from central and southern Myanmar. Zootaxa.

[3] Idiiatullina S.S., Pawangkhanant P., Tawan T., Worranuch T., Dechochai B., Suwannapoom C., Nguyen T.V., Chanhome L., Poyarkov N.A. (2023). Limestone jewel: A new colourful karst-dwelling pitviper (Serpentes: Viperidae: *Trimeresurus*) from the poorly explored borderlands of southern Peninsular Thailand. Vertebr. Zool..

[4] Idiiatullina S.S., Nguyen T.V., Pawangkhanant P., Suwannapoom C., Chanhome L., Mirza Z.A., David P., Vogel G., Poyarkov N.A. (2024). An integrative taxonomic revision of the *Trimeresurus popeiorum* species complex (Reptilia: Serpentes: Viperidae), with descriptions of two new species from the Indo-Burma biodiversity hotspot. Vertebr. Zool..

[5] Idiiatullina S.S., Nguyen T.V., Bragin A.M., Pawangkhanant P., Le D.X., Vogel G., David P., Poyarkov N.A. (2024). A new species of green pitviper of the *Trimeresurus macrops* complex (Reptilia: Serpentes: Viperidae) from South Central Coast Region of Vietnam. Zootaxa.

[6] Idiiatullina S.S., Pawangkhanant P., Suwannapoom C., Tawan T., Chanhome L., Nguyen T.V., David P., Vogel G., Poyarkov N.A. (2024). Another new species of karst-associated pitviper (Serpentes: Viperidae: *Trimeresurus*) from the Isthmus of Kra, Peninsular Thailand. Eur. J. Taxon..

[7] Pawangkhanant P., Idiiatullina S.S., Nguyen T.V., Ruangsuwan T., Matsukoji T., David P., Suwannapoom C., Poyarkov N.A. (2025). A snake can change its finery: A new cryptic species of the *Trimeresurus kanburiensis* complex (Reptilia: Serpentes: Viperidae) from Central Thailand with an unusual ontogenetic color change. Zootaxa.

[8] Gumprecht A., Tillack F., Orlov N.L., Captain A., Ryabov S. (2004). Asian Pit Vipers.

[9] Poyarkov N.A., Nguyen T.V., Popov E.S., Geissler P., Pawangkhanant P., Neang T., Suwannapoom C., Ananjeva N.B., Orlov N.L. (2023). Recent progress in taxonomic studies, biogeographic analysis, and revised checklist of reptiles in Indochina. Russ. J. Herpetol..

[10] David P., Teynié A., Vogel G. (2023). The Snakes of Laos.

[11] Rathee Y.S., Purkayastha J., Lalremsanga H.T., Dalal S., Biakzuala L., Muansanga L., Mirza Z.A. (2022). A new cryptic species of green pitviper of the genus *Trimeresurus* Lacépède, 1804 (Serpentes: Viperidae) from northeast India. PLoS ONE.

[12] Nguyen T.V., Idiiatullina S.S., Oo W.P., Lee J.L., Poyarkov N.A., David P., Vogel G. (2024). Range extension and expanded description of *Trimeresurus caudornatus* Chen, Ding, Vogel & Shi, 2020 (Serpentes: Viperidae), with the first country record from Myanmar. Zootaxa.

[13] Malhotra A., Thorpe R.S. (2004). A phylogeny of four mitochondrial gene regions suggests a revised taxonomy for Asian pitvipers (*Trimeresurus* and *Ovophis*). Mol. Phylogenetics Evol..

[14] Nguyen T.V., David P., Ananjeva N.B., Poyarkov N.A., Vogel G. (2025). A range extension of *Trimeresurus mayaae* Rathee et al., 2022 (Serpentes: Viperidae), with remarks on its pholidosis, natural history, and conservation status. Russ. J. Herpetol..

[15] Che J., Jiang K., Yan F., Zhang Y.P. (2020). Amphibians and Reptiles in Tibet: Diversity and Evolution.

[16] Xu W., Dong W., Fu T., Gao W., Lu C., Yan F., Wu Y., Jiang K., Jin J., Chen H. (2020). Herpetological phylogeographic analyses support a Miocene focal point of Himalayan uplift and biological diversification. Natl. Sci. Rev..

[17] Nguyen H.N., Tran B.V., Nguyen L.H., Neang T., Yushchenko P.V., Poyarkov N.A. (2020). A new species of *Oligodon* Fitzinger, 1826 from the Langbian Plateau, southern Vietnam, with additional information on *Oligodon annamensis* Leviton, 1953 (Squamata: Colubridae). PeerJ.

[18] Burbrink F.T., Lawson R., Slowinski J.B. (2000). Mitochondrial DNA phylogeography of the polytypic North American rat snake (*Elaphe obsoleta*): A critique of the subspecies concept. Evolution.

[19] Salvi D., Harris D.J., Kaliontzopoulou A., Carretero M.A., Pinho C. (2013). Persistence across Pleistocene ice ages in Mediterranean and extra-Mediterranean refugia: Phylogeographic insights from the common wall lizard. BMC Evol. Biol..

[20] Burland T.G. (2000). DNASTAR’s Lasergene sequence analysis software. Methods Mol. Biol..

[21] Creer S., Malhotra A., Thorpe R.S. (2003). Assessing the phylogenetic utility of four mitochondrial genes and a nuclear intron in the Asian pit viper genus *Trimeresurus*: Separate, simultaneous, and conditional data combination analyses. Mol. Biol. Evol..

[22] Malhotra A., Thorpe R.S. (2004). Maximizing information in systematic revisions: A combined molecular and morphological analysis of a cryptic green pitviper complex (*Trimeresurus stejnegeri*). Biol. J. Linn. Soc..

[23] Guo P., Liu Q., Zhong G., Zhu F., Yan F., Tang T., Xiao R., Fang M., Wang P., Fu X. (2015). Cryptic diversity of green pitvipers in Yunnan, South-west China (Squamata: Viperidae). Amphib. Reptil..

[24] Elangbam P.S., Biakzuala L., Shinde P., Decemson H.T., Vabeiryureilai M., Lalremsanga H.T. (2023). Addition of four new records of pit vipers (Squamata: Crotalinae) to Manipur, India. J. Threat. Taxa.

[25] Li J.N., Liang D., Wang Y.Y., Guo P., Huang S., Zhang P. (2020). A large-scale systematic framework of Chinese snakes based on a unified multilocus marker system. Mol. Phylogenetics Evol..

[26] Guo P., Liu Q., Zhu F., Zhong G.H., Chen X., Myers E.A., Che J., Zhang L., Ziegler T., Nguyen T.Q. (2016). Complex longitudinal diversification across South China and Vietnam in Stejneger’s pit viper, *Viridovipera stejnegeri* (Schmidt, 1925) (Reptilia: Serpentes: Viperidae). Mol. Ecol..

[27] Dawson K., Malhotra A., Thorpe R.S., Guo P., Mrinalini, Ziegler T. (2008). Mitochondrial DNA analysis reveals a new member of the Asian pitviper genus *Viridovipera* (Serpentes: Viperidae: Crotalinae). Mol. Phylogenetics Evol..

[28] Captain A., Deepak V., Pandit R., Bhatt B., Athreya R. (2019). A new species of pitviper (Serpentes: Viperidae: *Trimeresurus* Lacépède, 1804) from West Kameng District, Arunachal Pradesh, India. Russ. J. Herpetol..

[29] Guo P., Wang Y.Z. (2011). A new genus and species of cryptic Asian green pitviper (Serpentes: Viperidae: Crotalinae) from southwest China. Zootaxa.

[30] Sanders K.L., Malhotra A., Thorpe R.S. (2006). Combining molecular, morphological and ecological data to infer species boundaries in a cryptic tropical pitviper. Biol. J. Linn. Soc..

[31] Chen Z.N., Zhang L., Shi J.S., Tang Y.Z., Guo Y.H., Song Z.B., Ding L. (2019). A new species of the genus *Trimeresurus* from Southwest China (Squamata: Viperidae). Asian Herpetol. Res..

[32] Malhotra A., Thorpe R.S. (2000). A phylogeny of the *Trimeresurus* group of pit vipers: New evidence from a mitochondrial gene tree. Mol. Phylogenetics Evol..

[33] Chen Z.N., Yu J.P., Vogel G., Shi S.C., Song Z.B., Tang Y.Z., Yang J., Ding L., Chen C.S. (2020). A new pit viper of the genus *Trimeresurus* (Lacépède, 1804) (Squamata: Viperidae) from Southwest China. Zootaxa.

[34] Kumar S., Stecher G., Li M., Knyaz C., Tamura K. (2018). MEGA X: Molecular Evolutionary Genetics Analysis across computing platforms. Mol. Biol. Evol..

[35] Nguyen L.T., Schmidt H.A., Haeseler A.V., Minh B.Q. (2015). IQ-TREE: A fast and effective stochastic algorithm for estimating maximum likelihood phylogenies. Mol. Biol. Evol..

[36] Kalyaanamoorthy S., Minh B.Q., Wong T.K.F., von Haeseler A., Jermiin L.S. (2017). ModelFinder: Fast model selection for accurate phylogenetic estimates. Nat. Methods.

[37] Zhang D., Gao F.L., Jakovlić I., Zou H., Zhang J., Li W.X., Wang G.T. (2020). PhyloSuite: An integrated and scalable desktop platform for streamlined molecular sequence data management and evolutionary phylogenetics studies. Mol. Ecol. Resour..

[38] Hoang D.T., Chernomor O., Haeseler A.V., Minh B.Q., Vinh L.S. (2018). UFBoot2: Improving the ultrafast bootstrap approximation. Mol. Biol. Evol..

[39] Guindon S., Dufayard J.-F., Lefort V., Anisimova M., Hordijk W., Gascuel O. (2010). New algorithms and methods to estimate maximum-likelihood phylogenies: Assessing the performance of PhyML3.0. Syst. Biol..

[40] Ronquist F., Teslenko M., Van Der Mark P., Ayres D.L., Darling A., Höhna S., Larget B., Liu L., Suchard M.A., Huelsenbeck J.P. (2012). MrBayes 3.2: Efficient Bayesian phylogenetic inference and model choice across a large model space. Syst. Biol..

[41] Huelsenbeck J.P., Ronquist F., Nielsen R., Bollback J.P. (2001). Bayesian inference of phylogeny and its impact on evolutionary biology. Science.

[42] Dowling H.G. (1951). A proposed standard system of counting ventrals in snakes. Br. J. Herpetol..

[43] Darko Y.A., Voss O., Uetz P. (2022). A dictionary of abbreviations used in reptile descriptions. Zootaxa.

[44] Maki M. (1931). A monograph of the snakes in Japan.

[45] Pope C.H. (1935). The Reptiles of China. Turtles, Crocodilians, Snakes, Lizards.

[46] Zhao E.M., Huang M.H., Zong Y. (1998). Fauna Sinica: Reptilia. Volume 3. Squamata, Serpentes.

[47] David P., Captain A., Bhatt B.B. (2001). On the occurrence of *Trimeresurus medoensis* Djao in Djao and Jiang, 1977 (Serpentes: Viperidae: Crotalinae) in India, with a redescription of this species and notes on its biology. Hamadryad.

[48] David P., Vidal N., Pauwels O.S.G. (2001). A morphological study of Stejneger’s pitviper *Trimeresurus stejnegeri* (Serpentes: Viperidae: Crotalinae) with the description of a new species from Thailand. Russ. J. Herpetol..

[49] David P., Vogel G., Pauwels O.S.G., Vidal N. (2002). Description of a new species of the genus *Trimeresurus* from Thailand, related to *Trimeresurus stejnegeri* Schmidt, 1925 (Serpentes: Crotalidae). Nat. Hist. J. Chulalongkorn Univ..

[50] Ao J.M., David P., Bordoloi S., Ohler A. (2004). Notes on a collection of snakes from Nagaland, northeast India, with 19 new records for this state. Russ. J. Herpetol..

[51] Orlov N.L., Ryabov S.A., Bui T.N., Ho C.T. (2004). A new species of *Trimeresurus* (Ophidia: Viperidae: Crotalinae) from the karst region in central Vietnam. Russ. J. Herpetol..

[52] David P., Mathew R. (2005). Notes on some noteworthy snake specimens deposited in the collections of Eastern Regional Station of the Zoological Survey of India. Rec. Zool. Surv. India Occas. Pap..

[53] Guo P., Malhotra A., Thorpe R.S., Creer S., Pook C.E. (2009). Comments on the systematic status of specimens belonging to the genus *Viridovipera* (Serpentes: Viperidae: Crotalinae) from Sichuan and Yunnan provinces of southwestern China, with a redescription of *V. yunnanensis*. Herpetol. J..

[54] Teynié A., David P. (2014). Amphibiens et reptiles des formations karstiques du Laos [Amphibians and reptiles of the karst formations of Laos]. Bull. Société Herpétologique Fr..

[55] Nguyen T.Q., Nguyen T.V., Pham C.T., Ong A.V., Ziegler T. (2018). New records of snakes (Squamata: Serpentes) from Hoa Binh Province, northwestern Vietnam. Bonn. Zool. Beiträge.

[56] Li P.P., Zhao E.M., Dong B.J. (2010). Amphibians and Reptiles of Tibet.

[57] Huang K., Shi S.C., Qi Y., Wu J.Y., Yao Z.Y. (2021). *Lycodon gongshan* found in Tibet, China, with description of hemipenes and a new color type. Chin. J. Zool..

[58] Shi J.S., Liu J.C., Giri R., Owens J.B., Santra V., Kuttalam S., Selvan M., Guo K.J., Malhotra A. (2021). Molecular phylogenetic analysis of the genus *Gloydius* (Squamata: Viperidae: Crotalinae), with description of two new alpine species from the Qinghai–Tibet Plateau, China. ZooKeys.

[59] David P., Campbell P.D., Deuti K., Hauser S., Luu V.Q., Nguyen T.Q., Orlov N., Pauwels O.S.G., Scheinberg L., Sethy P.G.S. (2022). On the distribution of *Gonyosoma prasinum* (Blyth, 1854) and *Gonyosoma coeruleum* Liu, Hou, Ye Htet Lwin, Wang & Rao, 2021, with a note on the status of *Gonyosoma gramineum* Günther, 1864 (Squamata: Serpentes: Colubridae). Zootaxa.

[60] Guo K.J., Wu N.F., Shu F., Tang Z.J., Zhang T., Pubu D.Z., Chen S.D., Rao D.Q. (2022). *Elaphe carinata* (Günther, 1864) found in Chayu County, Tibet Autonomous Region. Sichuan J. Zool..

[61] Ren J.L., Jiang K., Huang J.J., David P., Li J.T. (2022). Taxonomic review of the genus *Herpetoreas* (Serpentes: Natricidae), with the description of a new species from Tibet, China. Diversity.

[62] Guo P., Liu Q., Jin J.-Q., Shu F., Wu Y.-Y., Che J. (2024). Multilocus molecular phylogeny and morphological comparisons provide new insights into systematics of the genus *Trachischium* Günther, 1858 (Serpentes: Natricidae). Curr. Herpetol..

[63] Guo P., Che J. (2024). Snakes in Qinghai–Xizang Plateau.

[64] Shu F., Lyu B., Guo K.J., Zhang T., Mi X.Q., Li L., Wu Y.Y., Guo P. (2024). Rediscovery of *Lycodon gammiei* (Blanford, 1878) (Serpentes: Colubridae) in Xizang, China, with comments on its systematic position. ZooKeys.

[65] Weng S.Y., Yang D.C., Wei C., Li P., Cui Z.B., Jiang Z.H., Huang S. (2024). A new record and a novel morph description of *Boiga stoliczkae* (Squamata: Colubridae) from China. Biodivers. Data J..

[66] Bohra S.C., Nguyen T.V., Vogel G., Lalremsanga H.T., Biakzuala L., Das M., Warjri H., Thongni G., Poyarkov N.A., Purkayastha J. (2025). Same but different: A systematic reassessment of *Hebius khasiensis* Boulenger, 1890 (Reptilia: Squamata: Natricidae) species complex from the Indo-Myanmar biodiversity hotspot supports the revalidation of *Natrix gilhodesi* Wall, 1925 as a valid species. Zootaxa.

[67] Jiang K., Lyu Z.T., Ren J.L., Li J.T. (2025). Updated checklist of serpents (Reptilia, Squamata) in China. Asian Herpetol. Res..

[68] Jiang K., Wu D.H., Huang J.J., Ren J.L., Gao Z.Y., Lyu Z.T., Li J.T. (2025). Description of a new species of kukri snake (Serpentes: Colubridae: *Oligodon*) from Xizang, China. Asian Herpetol. Res..

[69] Nguyen T.V., Le J.L., Jiang K., Ding L., Chit M.T., Poyarkov N.A., Vogel G. (2025). A new species of wolf snake *Lycodon* Fitzinger, 1826 from China and Myanmar (Squamata: Colubridae), and new data on *Lycodon gongshan* Vogel & Luo, 2011. Zootaxa.

[70] Patel H., Bhardwaj V.K., Thackeray T., Campbell P.D., Mirza Z.A. (2025). Phylogeny and systematics of *Oligodon juglandifer* (Wall, 1909) with comments on the taxonomic status of *Oligodon lipipengi* Jiang, Wang, Li, Ding, Ding & Che in Che et al. 2020. Diversity.

[71] Ren J.L., Huang J.J., Wu W., Jiang K., Li J.T. (2025). One mountain, two tigers: A new species of *Gloydius* (Serpentes: Viperidae) from the upper Lancang Valley in Xizang, China, with comments on the diagnostic characters and evolution of the *G. strauchi* (Bedriaga, 1912) species complex. Asian Herpetol. Res..

